# Therapeutic Targeting of Signaling Pathways Related to Cancer Stemness

**DOI:** 10.3389/fonc.2020.01533

**Published:** 2020-08-26

**Authors:** Asunción Espinosa-Sánchez, Elisa Suárez-Martínez, Laura Sánchez-Díaz, Amancio Carnero

**Affiliations:** ^1^Instituto de Biomedicina de Sevilla (IBIS), Hospital Universitario Virgen del Rocío, Universidad de Sevilla, Consejo Superior de Investigaciones Científicas, Seville, Spain; ^2^CIBER de Cancer, Madrid, Spain

**Keywords:** CSC, cancer, EMT, stem cell-like pathways, therapy

## Abstract

The theory of cancer stem cells (CSCs) proposes that the different cells within a tumor, as well as metastasis deriving from it, are originated from a single subpopulation of cells with self-renewal and differentiation capacities. These cancer stem cells are supposed to be critical for tumor expansion and metastasis, tumor relapse and resistance to conventional therapies, such as chemo- and radiotherapy. The acquisition of these abilities has been attributed to the activation of alternative pathways, for instance, WNT, NOTCH, SHH, PI3K, Hippo, or NF-κB pathways, that regulate detoxification mechanisms; increase the metabolic rate; induce resistance to apoptotic, autophagic, and senescence pathways; promote the overexpression of drug transporter proteins; and activate specific stem cell transcription factors. The elimination of CSCs is an important goal in cancer therapeutic approaches because it could decrease relapses and metastatic dissemination, which are main causes of mortality in oncology patients. In this work, we discuss the role of these signaling pathways in CSCs along with their therapeutic potential.

## Introduction

The understanding on the tumorigenesis process has been explained during decades according to the clonal evolution model. This model postulates that all cells within a tumor contribute to the maintenance of the tumor at different levels (1). In the cell, a number of genetic and epigenetic changes accumulate during time, and, by selection, the most aggressive cancer cells drive tumor progression (1, 2). Therefore, any cancer cell can become highly malignant, contributing to metastases and the resistance against therapies (2). However, currently, the cancer stem cell (CSC) model proposes a more suitable explanation to cancer complexity. The tumors contain a subset of different tumor cells, called cancer stem cells that are crucial for tumor initiation, progression, and recurrence (3, 4). These CSCs, through self-renewal and differentiation, are critical for the generation of most tumor cell types contributing to tumor heterogeneity. However, the rest of mature cells compose the bulk of the tumor, but are not responsible for the tumor generation. Therefore, tumor resistance to therapies and metastases are the direct result of these CSCs (5).

CSCs, or “tumor-initiating cells,” have the ability to self-renew and differentiate as normal stem cells. However, the mechanisms that regulate these processes are deregulated; therefore, CSCs continuously expand and produce differentiated progeny (5–7). Furthermore, CSCs can form new form tumors when grown into animals, but normal stem cells are unable to do (8, 9). CSCs compose a small population of cells within a tumor, share similar surface markers with normal stem cells (10, 11) and share common signaling pathways with normal stem cells (12, 13).

The size of the CSC pool is different in each tumor (14–16) and show, in general, a good correlation with agresivity and response, as well as patient survival (17, 18). CSCs show great heterogeneity, and may be different between grades and/or stages of the same type of tumors. The different pools of CSCs usually coexist in the same tumor, even within the same microenvironment niche. Interestingly, CSC different pools may communicathe physiologically among them thorugh phenotype interconversion (10, 19–21). CSCs are a “status” rather than a fixed “category” of cells. CSCs and non-CSCs can also interconvert in dynamic equilibrium. Non-CSCs can acquire CSC properties through de-differentiation (8, 22–24), and in this process, either genetic or epigenetic alterations, as well as microenvironment may be involved (25–28). Therefore, the CSCs model should be considered to be bidirectional, switching between stem and mature cells within the tumor (8, 22–24). Numerous studies support the concept that mature tumor cells (non-CSCs) are respond to cancer therapy, while CSCs are resistant to treatment [(29) and references therein]. In tumor stem cells (CSCs), different clonal evolution at the genetic and epigenetic levels generate distinct tumorigenic potential and heterogeneity which greatly influences disease progression and response to treatment (27, 29). On the other hand, the dynamic equilibrium between CSCs and differentiated non-CSCs adds another level of complexity. The potential for non-CSCs to revert to CSCs due to genetic, epigenetic, or microenvironment alterations that confer phenotypic plasticity to the tumor cell population is a strong driving force of tumor evolution and resistance to cancer therapy. Moreover, exposure of differentiated cells to therapeutic doses of radiation or many therapeutic compounds increases the CSC pool. To explain this CSC plasticity through cell maturation and dedifferentiating processes, it has been described that acquiring new genetic mutations, epigenetic changes, or microenviromental conditions that are able to activate the epithelial mesenchymal transition (EMT) induces the mature non-CSCs to dedifferentiate and acquire the CSC phenotype. Thus, activating TGF-β activates the EMT transcription factors Twist or Snail, and/or hypoxic conditions seem to influence dedifferentiation processes greatly, activating the SC pathways in tumor mature cells, leading to new CSCs (21, 30–33). Therefore, the phenotypic plasticity inducing conversions between mature non-CSCs and CSCs influences tumor evolution and clinical management.

The capability of one cell type to acquire the phenotype of another or of differentiated somatic and stem cells to interconvert states is denominated *cellular plasticity* (22, 34). This plasticity may explain the altered gene expression found in different tumor types resembling cell lineages that differ from the true progenitors (22, 35–38). Indeed, the inherent plasticity of stem cell pathways such as Wnt, Notch or Hedgehog, can be modified suggesting that these pathways may be relevant for anticancer research (5, 34, 39–41).

**Figure 1:**
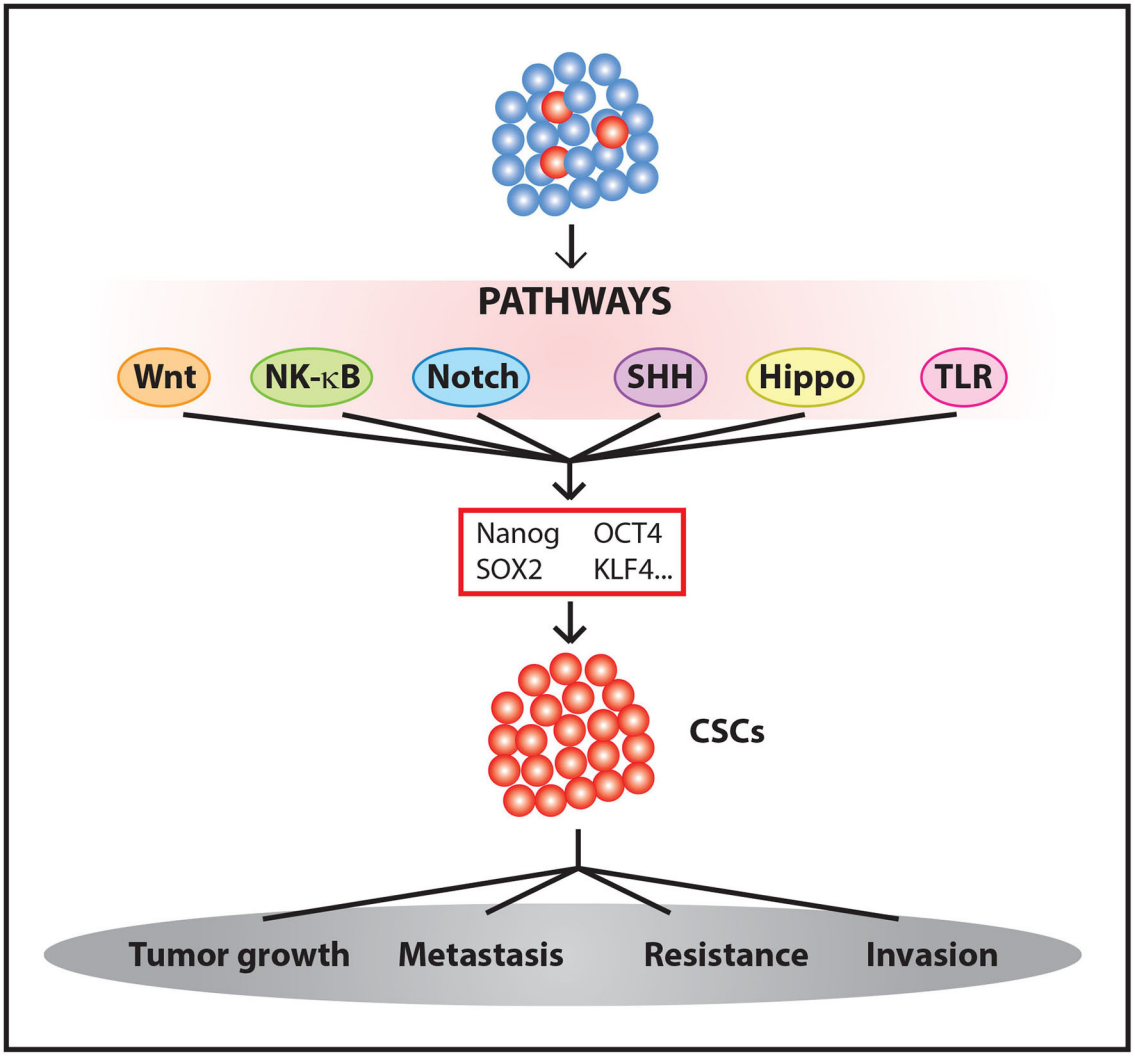
Generation and maintenance CSCs. The activation of different signaling pathways leads to Yamanaka factors expression among other genes, promoting the enrichment of CSC populations within the tumor.

These and other results suggest that some oncogenic signals are able to induce CSCs. These signals are accompanied by an increase in resistance to chemotherapeutic treatments (35, 36) and, in some cases, radiotherapy (42, 43). Therefore, we must take into account the processes involved in the activation of stemness pathways and tumor evolution and evaluate how their influences affect therapy to effectively eliminate a tumor (Figure 1).

Therefore, cancer cells can move from stem to differentiated states, and viceversa, in response to therapy, transcription changes or signaling in the microenvironment (20, 44, 45). Moreover, inside a single tumor, CSCs can coexist in more than one metabolic and/or pluripotency state. CSCs from breast cancer, for example, can be found in different mesenchymal- and epithelial-like states (24, 46). The transition between these states has been reported to be regulated by epigenetic alterations (47). Phenotypic plasticity contributes to the complexity of the cancer ecosystem and represents a major challenge for tumor eradication since it actively contributes to tumor cell survival and metastasis.

CSC cells present many mechanisms for therapy resistance, such as high-level of drug efflux pumps, reactive oxygen species scavengers, antiapoptotic proteins, DNA repair efficient mechanisms, interactions with the protective microenvironment (37, 48–51) or exosomes loaded with proteins of non-coding RNA prone to modify the environment to favor metastasis (51–54). On the other hand, similar to normal stem cells, CSCs are known to be slow cycling in many tumors and are maintained in the G0 phase (55).

Epigenetic mechanisms may mediate therapeutic resistance in CSCs in many different ways (27, 35, 43, 51, 56–59). The silencing of the epigenome is also involved in maintaining plasticity and the transition of mature tumor non-CSCs to CSCs, as reported for the transition of metabolic states in renal tumor cells by the inactivation of MYBBP1a and the activation of MYB (60–62). For example, epigenetic demethylation of MAP17 driving the resistance against some targeted therapies was observed in lung adenocarcinoma (43). Additionally, studying lung cancer, Sharma and coworkers reported that a reversible drug-tolerant state of EGFR TKi therapy was obtained by chromatin alterations induced by histone demethylase activity (63). These and other results established that CSCs can regulate epigenetic factors to maintain their pool and overcome targeted therapies. However, the reversible nature of these epigenetic alterations suggests that inhibitors of the pathways modifying these epigenetic regulators may hold promise as relevant clinical therapeutic targets, either alone or in combination.

Thus, the CSC hierarchical model explains the failure of treatment and tumor recurrence and promises new targets for anticancer drug discovery. This article does not pretend to be an exhaustive review of all CSC pathways related to plasticity and/or therapeutic approaches. We summarize some evolving treatment strategies related to these pathways with the aim of shedding new light on current therapy development with promising new anticancer agents. Other CSC-related signaling pathways more commonly studied, not reviewed here, but relevant to stemness include MYB, TGF-β, JAK-STAT, FGFs, PI3K, or MEK. Targeting these pathways has been shown to exert anti-CSC effects, and promising agents are currently under investigation, as recently reviewed elsewhere (30, 36, 37, 47, 48, 51, 62, 64–74).

## Wnt Pathway

The Wnt pathway is involved in embryonic development and homeostasis. Moreover, this signaling pathway regulates cell proliferation and survival (75). This pathway has two different signaling pathways: canonical (β-catenin dependent) and non-canonical (β-catenin-independent).

**Figure 2:**
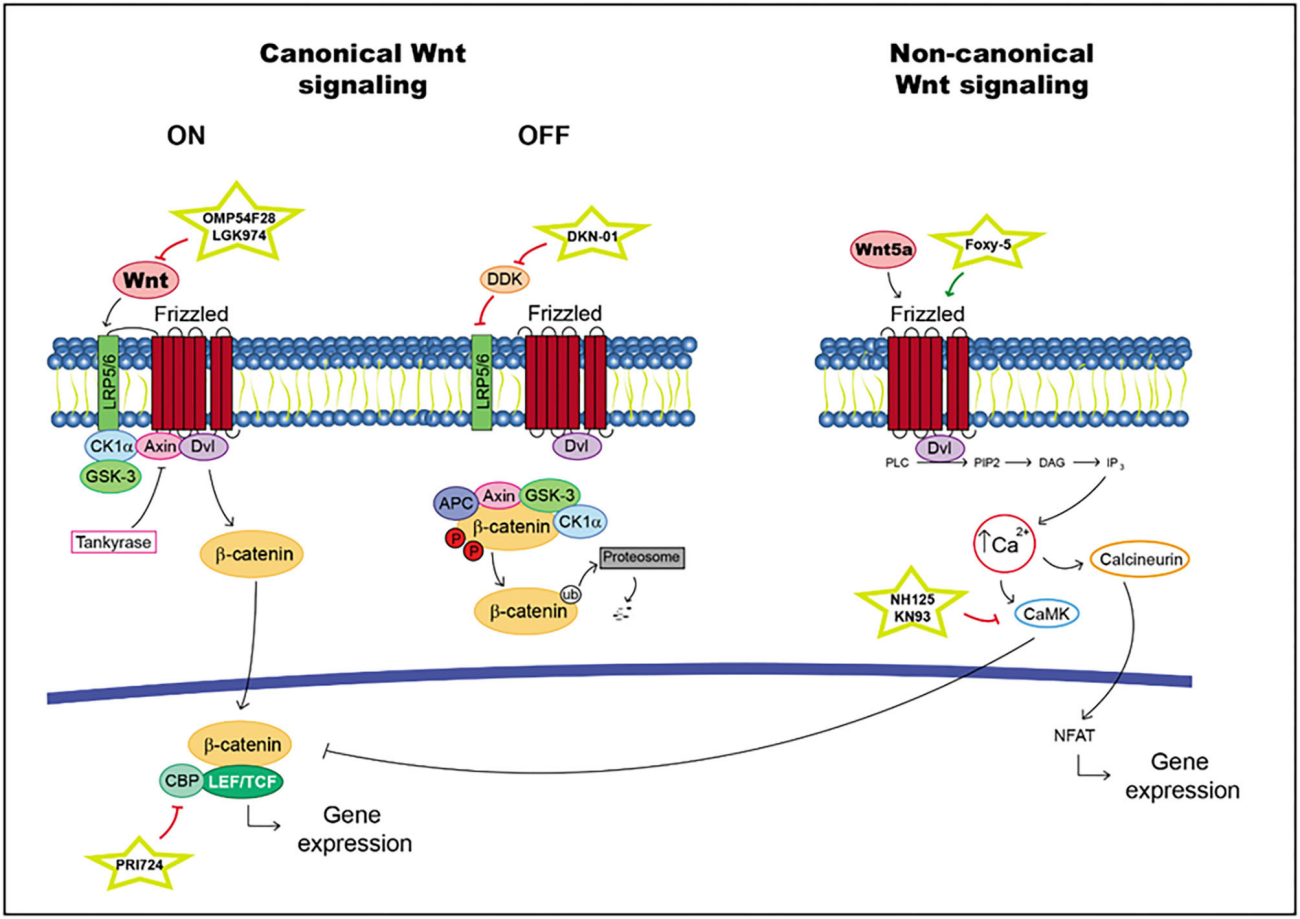
Wnt pathway including targets and drugs that could be employed to inhibit it. **(Left)** canonical Wnt signaling. **(Right)** non-canonical Wnt signaling.

According to canonical signaling, Wnt is secreted and binds to Frizzled receptors and/or the low-density lipoprotein-related protein (LRP) 5 and 6 coreceptors. When R-spondin ligands bind to Lgrs, the action of the Wnt pathway is enhanced. In the presence of a Wnt ligand, Wnt interacts with Frizzled and/or LRP5/6 (76). This situation generates a cascade in which the phosphorylation of the cytoplasmic domain in LRP, the recruitment of scaffolding protein Disheveled (Dvl) and the hijack of GSK-3b and Axin are realized, with the last molecule produced through tankyrases. Thus, β-catenin is available for translocation into the nucleus, where it binds to lymphoid enhancer factor (LEF)/T cell factor (TCF) transcription factors and activates target gene transcription (76). In the absence of a Wnt ligand, the level of intracellular β-catenin is very low due to the action of the proteasome degradation complex; this complex is composed of scaffolding protein Axin and adenomatous polyposis coli (APC). Moreover, the kinase proteins glycogen synthase kinase-3b (GSK-3b) and casein kinase 1a (Ck1a) phosphorylate β-catenin triggering its ubiquitination (76) (Figure 2).

In non-canonical signaling, transduction proceeds in some different ways; for example, in the Wnt/Ca^2+^ pathway. The interaction between Wnt5A and Frizzled FZD2 receptors activates Dvl. Moreover, it promotes the release of Ca^2+^ into the cytosol as facilitated by FZD2, which cleaves guanine nucleotide-binding protein (G protein). Ca^2+^ activates CaMKII_AD (Ca^2+^/calmodulin-dependent protein kinase II) and suppresses the canonical Wnt pathway. This situation increases the differentiation of cells in the neuronal system (77–80) (Figure 2).

### Cancer Stem Cells

Some articles suggest that the role of Wnt in the regulation of CSCs differs depending on the types of cancer, such as blood, intestine, lung, mammary, gland, nervous system, skin, and urinary tract. During the development and maintenance of CSCs, the most common alteration is the hyperactivation of Wnt signaling. In skin cancer, the role of the Wnt pathway was discovered using a reporter mouse strain. It was observed that the genetic deletion of β-catenin produced induced tumor regression in chemically induced skin tumors through a decrease in the pool of CD34+ stem cells (81). In human acute leukemia, the crucial role of Wnt signaling activation in cell self-renewal capacity and drug-resistant properties has been suggested (82). Another article reported that the overexpression of miR-582-3p causes Wnt signaling activation by targeting multiple negative regulators of the Wnt pathway, such as AXIN2, DKK3, and SFRP1. Therefore, miR-582-3p promotes tumorigenesis in NSCLC and CSCs *in vitro* (83). Wnt enhancer R-Spondin-2 (RSPO2) is highly expressed in subpopulations with high intrinsic Wnt activity and with properties indicative of CSCs. Therefore, the employment of promoter inhibitors such as RSPO2 was proposed to block stemness-promoting pathways (84). In metastatic colorectal cancer, an increase in the level of progastrin, a tumor-promoting peptide essential for the self-renewal of colon CSCs, was observed, implicating β-catenin/TCF4 as a direct target gene. Therefore, therapy employing antibodies directed against progastrin was proposed for patients with metastatic colorectal cancer K-RAS-mutations (85). A recent article suggested a role for SOX8 in cancer stem cell properties and therapy resistance. It reported that SOX8 conferred chemoresistance and enhanced stemness properties. Additionally, SOX8 mediated EMT via the FZD7-mediated Wnt/β-catenin pathway (86).

### Therapeutic Targets and Drugs

A large number of mutations in the Wnt pathway have been identified. Above all, aberrations in the expression of Wnt ligands, Frizzled receptors, β-catenin and APC were discovered in many different tumors (87–89). According to these results, this pathway has been researched in recent years to identify potential therapeutic targets. Some drugs that may be potential therapeutics to be employed in the treatment of different tumors are detailed.

A fusion protein called OMP-54F28, ipafricept (OncoMed Pharmaceuticals), competes for the FZD8 receptor and antagonizes Wnt signaling. Preclinical models with this drug have shown reduced tumor growth as a single drug in combination with other chemotherapeutic agents (90). OMP-54F28 was studied in phase Ib clinical trials for different types of cancer (ClinicalTrials.gov Identifiers: NCT02050178, NCT02069145, and NCT02092363). In ovarian cancer patients, ipafricept was well-tolerated in combination with sequential carboplatin/paclitaxel, but it caused bone toxicity at efficacy doses; therefore its use is limited (91).

Another drug of interest is SM08502 (Samumed LLC), which is a potent CDC-like kinase (CLK) inhibitor. Moreover, CLKs play important roles in the integral cellular mechanism of mRNA splicing to induce the retention of introns in DVL2, TCF7, ERBB2, and LRP5 and exon skipping in LEF1 and TCF7L2 (92). SM08502 inhibits the growth of gastrointestinal tumors in xenograft mouse models (93). The molecule was recently found in phase 1 (ClinicalTrials.gov Identifier: NCT03355066) to show safety, tolerability and efficacy in patients with advanced solid tumors.

On the other hand, the combination of gedatolisib (a pan-class I PI3K/mTOR isoform inhibitor) and PTK7-ADC (an antibody-drug conjugate used against the cell-surface PTK7 protein) has a double synergistic effect, whereby gedatolisib increases the expression of PTK7-ADC and an auristatin payload causes an effect similar to that of gedatolisib. Currently, this combination is in s phase I trial for patients with metastatic triple-negative breast cancer because the PI3K pathway is dysregulated in most cells of this type of cancer (94).

The inhibition of porcupine, an O-acyltransferase required for Wnt activation and secretion, induced by LGK974 (Novartis, Basel, Switzerland, https://www.novartis.com), reduced the phosphorylation of the LRP6 receptor and the expression of WNT target genes in preclinical models. Furthermore, *in vivo* studies of LGK974 have demonstrated an antitumoral response in different types of cancer (95). Phase I evaluation of LGK974 alone and in combination with PDR001 (spartalizumab, an anti-PD-1 antibody) is ongoing (ClinicalTrials.gov Identifier: NCT01351103) in patients with lobular or triple negative breast cancer, melanoma or pancreatic cancer (96).

A monoclonal antibody against dickkopf WNT signaling pathway inhibitor 1 (DKK1) and DKN-01 was discovered. The high expression of DKK1 may be a predictive biomarker for effective treatment with DKN-01 and pembrolizumab in gastroesophageal adenocarcinoma (97). A phase 2 study is assessing this molecule as a monotherapy or in combination with paclitaxel in patients with endometrial, uterine or ovarian cancer (ClinicalTrials.gov Identifier: NCT03395080).

PRI-724 (PRISM BioLab) is a small molecule that inhibits the interaction between β-catenin and CBP in the nucleus. Thus, the downregulation of genes involved in symmetric non-differentiation division and increased p300/β-catenin binding were produced. This situation promotes stem cell differentiation and increases the sensitivity of cytotoxic drugs (98). Phase IIb of this drug in combination with gemcitabine in patients with advanced pancreatic adenocarcinoma (APC) has been completed, and the results showed the necessity for more work to find useful predictive and PD markers.

Aberrant activation of signaling from Wnt pathway usually is associated with stem-like phenotypes and EMT, which induce resistance to endocrine therapy. Plumbagin inhibited colony formation and mammosphere formation and decreased the number of cancer stem cell markers by inhibiting the Wnt signaling pathway. Plumbagin also reduced Wnt-dependent genes as well as β-catenin. Moreover, treatment of orthotopic xenografts with Plumbagin inhibited tumor growth, angiogenesis and metastasis without significant adverse effects on body weight or blood coagulation; therefore, Plumbagin may be useful to treat endocrine-resistant breast cancer (99).

On the other hand, the inhibition of the non-canonical Wnt pathway can be achieved through different drugs. Foxy-5 is a mimetic Wnt5a molecule that binds to and activates the Frizzled 2/5 receptor. Increased Wnt-5a signaling may inhibit endothelial tumor cell migration and invasion. This effect may decrease the metastasis of susceptible tumor cells (100). A phase 2 evaluation of it as a neoadjuvant therapy will be performed for subjects with colon cancer who express low levels of Wnt-5a (ClinicalTrials.gov Identifier: NCT03883802). Moreover, other molecules, such as NH125, which is more selective as an eEF-2 kinase inhibitor that is as an PKC, PKA, or CaMKII inhibitor (101), and KN93, which is a selective inhibitor of CaMKII (102), may be potential drugs to regulate the non-canonical Wnt pathway (Table 1).

**Table 1:** Overview of drugs targeting CSC pathways.

**Pathway**	**Agent**	**Mechanism of action**	**Model**	**Clinical trial phase**
Wnt pathway	OMP-54F28	Wnt antagonist	Solid tumors	Phase Ib
	SM08502	CDC-like kinase (CLK) inhibitor	Advanced solid tumors	Phase I
	Gedatolisib (1) and PTK7-ADC (2)	(1) PI3K/mTOR inhibitor	Metastatic triple-negative breast cancer	Phase I
		(2) PTK7 protein antagonist		
	LGK974	Porcupine inhibitor	Melanoma, breast cancer, and pancreatic cancer	Phase I
	DKN-01	DKK1 inhibitor	Endometrial, uterine, and ovarian cancer	Phase II
	PRI-724	CBP inhibitor	Advanced pancreatic adenocarcinoma	Phase IIb
	Plumbagina	Wnt ligands inhibitor	Endocrine resistant breast cancer	Preclinical
	Foxy-5	Mimetic Wnt5a molecule	Colon cancer	Phase II
	NH125	eEF-2 kinase inhibitor	Solid tumors	Preclinical
	KN93	CaMKII inhibitor	Solid tumors	Preclinical
NFkB pathway	Thalidomide	TNF-a inhibitor	Solid tumors	Phase II
	Bortezomib	Proteosome inhibitor	Myeloma, lymphoma, and acute leukemia	Phase III
	BMS-345541	IKKb and IkB protein inhibitor	Lung cancer	Preclinical
	Bardoxolone methyl (RTA-402)	IKKb and JAK1 inhibitor	Pancreatic adenocarcinoma and lymphomas	Phase I
	IMD-0354 and KRT1853	IKKb inhibitor	Lung cancer	Preclinical
	PS1145	IKK inhibitor	Prostate cancer and nasopharyngeal carcinoma	Preclinical
Notch pathway	PF-03084014	γ-secretase inhibitor (GSI)	Desmoid tumors	Phase II/III
	BMS-906024	γ-secretase inhibitor (GSI)	T-ALL relapses and T cell lymphoblastic lymphoma	Phase I
			Adenoid cystic adenocarcinoma	Phase II
	MK-0752	γ-secretase inhibitor (GSI)	Recurrent central nervous system tumors, advanced breast cancer, and T-ALL	Phase I
	MK-0752 + docetaxel/gemcitabine	γ-secretase inhibitor (GSI)	Locally advanced or metastatic breast and pancreatic cancer, respectively	Phase I/II
	RO4929097	γ-secretase inhibitor (GSI)	Renal cell carcinoma, metastatic pancreatic cancer, and non-small cell lung cancer relapses	Phase II
	RO4929097 + temsirolimus/	γ-secretase inhibitor (GSI)	Solid tumors	Phase I
	Cediranib/gemcitabine/			
	Bevacizumab			
	Enoticumab	DLL-4 monoclonal antibody	Advanced solid cancers (ovarian carcinoma)	Phase I
	Demcizumab + gemcitabine	DLL-4 monoclonal antibody	Pancreatic cancer	Phase I
				
	Demcizumab + FOLFIRI	DLL-4 monoclonal antibody	Colorectal cancer	Phase I
	Demcizumab + Carboplatin and pemetrexed	DLL-4 monoclonal antibody	Non-squamous NSCLC	Phase II
	Tarextumab	Notch2, 3 monoclonal antibody	Epithelial cancers (breas, small cell lung, ovarian, and pancreatic cancers)	Phase I/II
	Brontictuzumab	Notch1 monoclonal antibody	Hematological malignances and advanced solid tumors	Phase I
	AMG 757	DLL-3 Bi-specific T cell engager	Neuroendocrine carcinomas (small cell lung cancers)	Phase I
	AMG 119	DLL-3 Chimeric antigen receptor (CAR) T cell	Neuroendocrine carcinomas (small cell lung cancers)	Phase I
	Rovalpituzumab tesirine	DLL-3 antibody-drug conjugate (ADC)	Neuroendocrine carcinomas (small cell lung cancers)	Phase II
	CB-103	Pan-Notch inhibitor against transcriptional complex	Advanced-stage solid tumors and hematological malignancies	Phase I/IIa
Sonic Hedgehog pathway	Vismodegib	SMO inhibitor	Metastatic basal cell carcinoma (BCC) or recurrent locally advanced BCC	Approved by FDA 2012 + clinical trials in other tumors (breast cancer)
	Sonidegib	SMO inhibitor	Recurrent locally advanced BCC	Approved by FDA 2015
	Saridegib	SMO inhibitor	Chondrosarcoma, head and neck, pancreatic, adenocarcinoma, BCC, myelofbrosis	Phase II
	BMS-833923	SMO inhibitor	Basal cell nevus syndrome chronic myeloid leukemia	Phase II
	Glasdegib	SMO inhibitor	Acute myeloid leukemia, other cancers	Phase II
	Taladegib	SMO inhibitor	Various carcinomas and sarcomas	Phase II
	TAK-441	SMO inhibitor	BCC, advanced non-hematologic malignancies	Phase I
	Arsenic trioxide	GLI1, 2 inhibitor	Acute promyelocytic leukemia	Approved by FDA 2000 + phase I–IV clinical trial in other tumors
	Genistein	GLI1 inhibitor	Different solid tumors (gastric, prostate, breast) and hematological malignancies	Phase I/II
	5,00E+01	SHH monoclonal antibody	Various cancer types	Preclinical
	RU-SKI43	SHHat enzyme inhibitor	Various cancer types	Preclinical
Hippo pathway	Verteporfin	YAP-TEAD interaction inhibitor	Metastatic breast cancer, pancreatic tumors, prostate cancer	Phase I–II
	CA3	YAP-TEAD interaction inhibitor	Esophageal adenocarcinoma	Preclinical
	Flufenamic acid	YAP-TEAD interaction inhibitor	Metastatic breast tumor and hepatocellular carcinoma	Preclinical
	Chloromethyl ketone	YAP-TEAD interaction inhibitor	Various cancer types	Preclinical
	Super-TDU	YAP-TEAD interaction inhibitor	Pancreatic, gastric, and colorrectal cancer	Preclinical
	CT-707	YAP post-transcriptional modifications	Non-small cell lung	Phase I
	C19	MST agonist	Melanoma, breast cancer, colon cancer, and neuroblastoma	Preclinical
	BET inhibitors	Epigenetic modulation	Solid and hematological malignancies	Phase I–II
	Panobinostat	Epigenetic modulation	Solid and hematological malignancies	Phase I–IV
TLR pathway	Bacillus Calmette-Guerin (BCG)	TLR2/4 agonist	Carcinoma *in situ* or muscle non-invasive cancer of the urinary bladder	FDA approved in 1998 + Clinical trials (melanoma, colorectal and lung cancer…)
	CADI-05	TLR2 agonist	Advanced melanoma	Phase I–II
	Monophosphoryl lipid A (MPLA)	TLR4 agonist	Adjuvant in vaccines of human papillomavirus (HPV)-associated cervical cancer	FDA approved + Phase I–IV in other tumors
	Glucopyranosyl lipid A	TLR4 agonist	Skin and colorectal cancer, sarcoma, lymphoma	Phase I–II
	AS15	TLR4 agonist	Various cancer types	Phase I–III
	Poly (I:C)	TLR3 agonist	Various cancer types	Phase I–II
	Poly-ICLC (Hiltonol®)	TLR3 agonist	Solid tumors	Phase I–II
	Rintatolimod (Ampligen®)	TLR3 agonist	Fallopian tube, ovarian, colorectal, prostate, and brain tumors	Phase I–II
	Entolimod (CBLB502)	TLR5 agonist	Local or metastatic malignancies	Phase I-II
	Imiquimod	TLR7/8 agonist	Superficial basal cell carcinoma	FDA approved + Phase I–IV in other tumors
	Resiquimod	TLR7/8 agonist	Skin tumors and vaccine adjuvant	Phase I–II
	852A	TLR7 agonist	Melanoma, breast, ovarian, endometrial, cervical and esophageal cancer, and hematological malignancies	Phase I–II
	VTX-2337	TLR8 agonist	Solid and hematological maliganancies	Phase I–II
	Agatolimod (CpG7909)	TLR9 agonist	Vaccine adjuvant in solid and hematological malignancies	Phase I–II
	SD-101	TLR9 agonist	Various solid tumors and lymphoma	Phase I–II
	E5564	TLR4 antagonist	Leukemia	Phase I
	CRX-526	TLR4 antagonist	Colon cancer	Preclinical
	OPN305	TLR2 antagonist antibody	Myelodysplastic syndrome	Phase I–II

## NF-κB Pathway

The NF-κB pathway is a complex and pathway that has been studied mainly for its controversial roles in inflammation and immune responses. This signaling also plays an important role in cellular proliferation, survival and differentiation (103–105).

The inflammatory effects on cancer development have been studied, above all the modulation of inflammation-associated cancer through the transcription factor NF-κB (106). The excessive innate immunity activation and growth caused by NF-κB are involved in tumor development and progression (107). Moreover, the activation of NF-κB could be caused for genetic alterations such as amplification, mutations, or deletions in different cancer cells. NF-κB binds to promoters of genes such as IL1B, TNF, and IL6 which cause the secretion of cytokines and chemokines (108). For this reason, it is suggested that the exposure to proinflammatory stimuli in tumor microenvironment or genetic alterations in components of IKK—NF-κB pathway could cause the activation of NF-κB in cancer.

The NF-κB family of transcription factors includes five different proteins: p105/p50, p100/p52, p65 (RelA), c-Rel, and RelB, which are inactivated in the cytoplasm upon binding to IκB proteins (109). Two signals are differentiated: canonical and non-canonical.

LPS and proinflammatory cytokines, such as IL-1, Toll-like receptors (TLRs) and tumor necrosis factor alpha (TNF-α), activate the NF-κB signaling pathway. The recruitment of a receptor proximal adaptor protein facilitates the phosphorylation and activation of IκB kinase (IKK) protein complex, which subsequently initiates the phosphorylation of IκB proteins. IκB proteins are degraded, allowing NF-κB translocation into the nucleus to activate specific target genes. This pathway can activate antiapoptotic factors and cytokines as well as proliferation factors (109).

**Figure 3:**
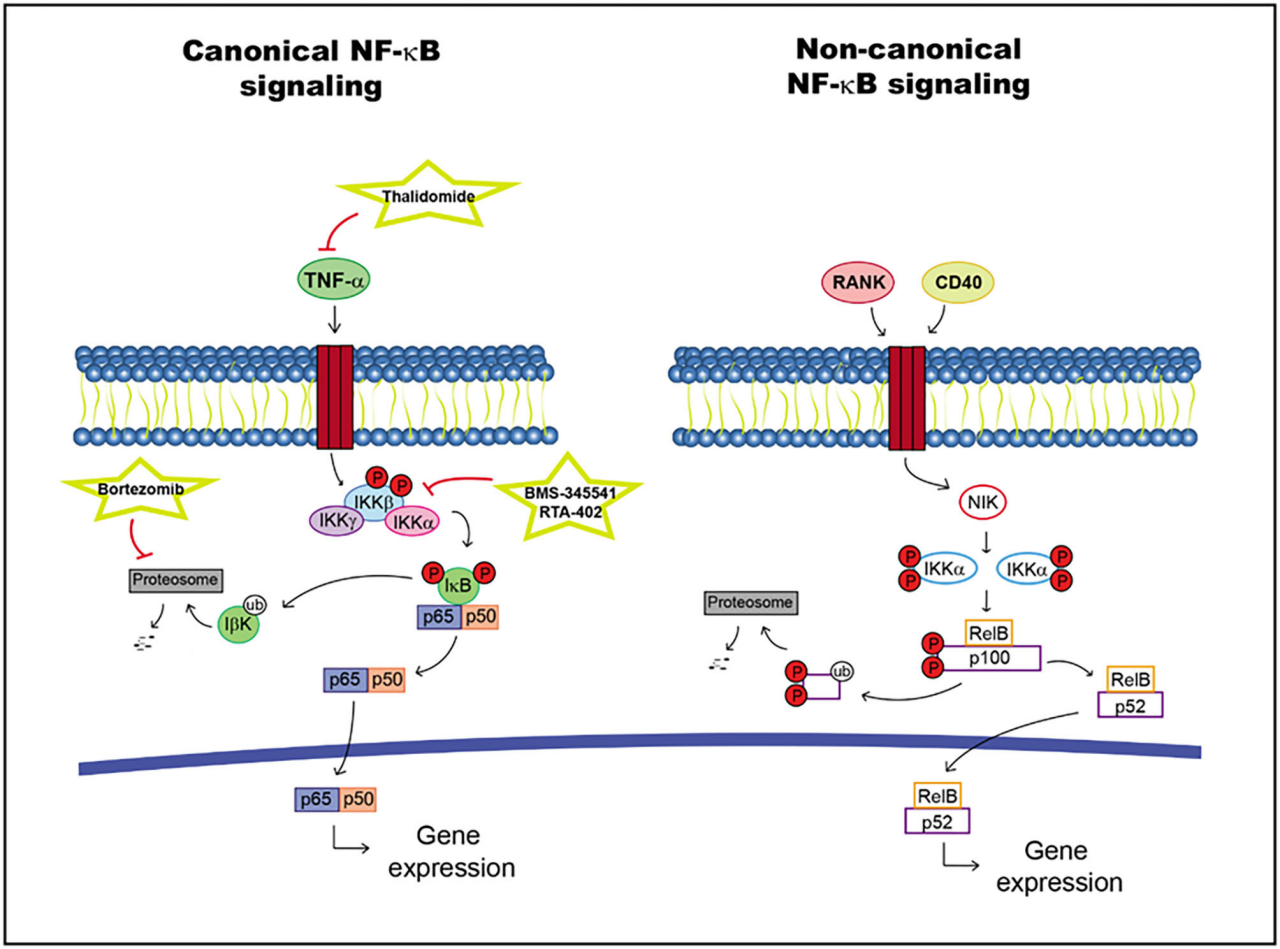
NF-κB pathway including targets and drugs that could be employed to inhibit it. **(Left)** canonical NF-κB signaling. **(Right)** non-canonical NF-κB signaling.

The non-canonical brand of the NF-κB pathway may activated by different factors such as the receptor activator of NF-κB (RANK) and CD40, leading to the stabilization of NF-κB-inducing kinase (NIK). This molecule activates IKKα dimers, promoting p100 phosphorylation and cleavage into p52. The activated RelB/p52 dimer can be translocated into the nucleus regulating specific target gene transcription (109, 110) (Figure 3).

### Cancer Stem Cells

The link between inflammation and cancer stem cells (CSCs) is found during tumorigenesis and disease progression (111, 112). The involvement of the NF-κB pathway in CSCs was discovered in primary AML samples, in which the DNA in CD34+ cells bind with NF-κB. However, this binding was not observed in normal hematopoietic stem cells (113). In adult murine neurogenesis, TLR2 and TLR4 were found to have opposite functions in adult neural stem/progenitor cell (NPC) proliferation and differentiation. Moreover, the inhibition of the LRPs impacted the self-renewal and the cell fate decision of NPCs (114). A MEC-targeted inducible transgenic inhibitor of NF-κB was developed in mammary oncomice, and it inhibited breast tumor stem cell markers and expanded NANOG and SOX2 expression *in vivo* and *in vitro* (115).

Chronic inflammation may be responsible for the accumulation of proinflammatory cytokines, which increase the action of NF-κB. This situation promotes a tumorigenic microenvironment in colon cancer (116). In the basal-like subtype of triple-negative breast cancer, high levels of activated NF-κB pathway components were observed. Inflammatory cytokines or dysregulated NIK expression induced this activation and caused the upregulation of JAG1 expression in normal cancer cells. NOTCH signaling was stimulated in cancer stem cells and induced the expansion of CSC populations (117). The upregulation of NIK caused a decrease in the CSC population, and its reduction led to the results that were opposite those found for breast cancer (118). In addition, mammary stem and progenitor cells were expanded by constitutive RANK signaling, and the overexpression of this activator increased tumorigenesis properties (119).

IKKα and its activator, NF-κB-inducing kinase, were critical for the expansion of tumor-initiating cells (TICs). IKKα is translocated into the nucleus, where it phosphorylates the cyclin-dependent kinase (CDK) inhibitor p27/Kip1 and promotes its nuclear export or exclusion (120).

### Therapeutic Targets and Drugs

To determine the efficacy of cancer treatment through the inhibition of the NF-κB pathway, various problems have been found: low efficacy of NF-κB inhibitors in addition to lymphoma and leukemia (121), immunosuppression after long-term systemic administration of these drugs (122) and the appearance of short-term drug resistance (123, 124). However, NF-κB inhibitors can be useful in combination with other chemotherapies, as most anticancer agents can activate this pathway, protecting cancer cells from apoptosis (125).

TNF-α is a target that can be employed to inhibit the NF-kB pathway. Drugs such as thalidomide and its derivatives act in this way (126, 127). However, thalidomide does not show good results in combination with gemcitabine or irinotecan chemotherapy, and its use increases the risk of thrombotic events (128).

On the other hand, bortezomib is an innovative drug with remarkable preclinical and clinical antitumor activity in different types of cancer. The mechanism of action consists of proteasome inhibition, which causes inactivation of the canonical NF-κB pathway (129, 130). Additionally, the prolonged inhibition of this pathway accelerates chemical lung carcinogenesis by perpetuating carcinogen-induced inflammation (131). Currently, many phase III clinical trials are in the recruitment stage for patients with myeloma, lymphoma or acute leukemia who will be treated with bortezomib in combination with other anticancer agents, and the results will be compared to those of conventional treatments.

There is another type of drug that inhibits IKKβ and IκB protein, BMS-345541, which causes the reduction of stemness, self-renewal and migration capacity in lung cancer (132). Another drug in this group, bardoxolone methyl (RTA-402), is a triterpenoid and antioxidant inflammation modulator that inhibits IKKβ and JAK1. Phase I studies have been conducted for various types of cancer: pancreatic adenocarcinoma (133), solid tumors and lymphomas (134). Additionally, IMD-0354 and KRT1853 are IKKβ inhibitors and block IκBα phosphorylation, inactivating its translocation into the nucleus and the activation of NF-κB (135). In lung cancer, it was discovered that these drugs can be potential anticancer agents through suppression of cancer cell invasion, proliferation, and survival (136). In addition, PS1145 reduced the growth of tumorigenic prostate cancer (137) and nasopharyngeal carcinoma (PNC) cell lines (138). Moreover, in the most recent study, it was observed that NF-κBp65 and KLF4 upregulation was involved in drug resistance (138).

These drug groups only partially inhibit NF-κB signaling because IKKα can also activate this pathway. Therefore, inhibitors of IKKα and IKKβ need to be found to generate better effects (139) (Table 1).

## Notch Pathway

The Notch pathway is an evolutionarily conserved signaling route involved in a variety of developmental and homeostatic processes, such as proliferation, stem cell maintenance, cell fate specification, differentiation, or angiogenesis, despite the apparent simplicity of its signaling network. The effects of activating this signaling pathway are very diverse, depending on the signal dose and cell context (140–144). Its deregulation contributes to a wide range of disorders and diseases, such as congenital afflictions, viral infections, and/or different types of cancer (142).

The Notch signaling pathway is composed of Notch receptors and Notch ligands, as well as different proteins that serve as posttranslational modifiers, but there is not an amplification cascade induced by different proteins in well-known signaling pathways (142, 145).

There are four Notch receptors encoded by different genes: Notch1–4. The general structure of Notch receptors consists of a single-pass transmembrane protein composed of different protein modules, with some differences: Notch3 and 4 have a shorter extracellular domain and lack the intracellular transcription-activating region, and Notch4 lacks the Notch cytokine response domain. Notch1 and Notch2 are expressed in many tissues during development and in adults, whereas Notch3 is expressed mostly in vascular smooth muscle and pericytes, and Notch4 is expressed in the endothelium (142, 145–149).

There are four Notch ligands in humans: Dll1 and Dll4, from the Delta family of ligands, and Jag1 and Jag2, from the Serrate family. There is a Dll3 ligand, but it is not able to activate Notch receptors. These ligands are also single-pass transmembrane proteins. The expression pattern of these ligands is less established than that of the Notch receptor ligands (142, 145, 150, 151).

**Figure 4:**
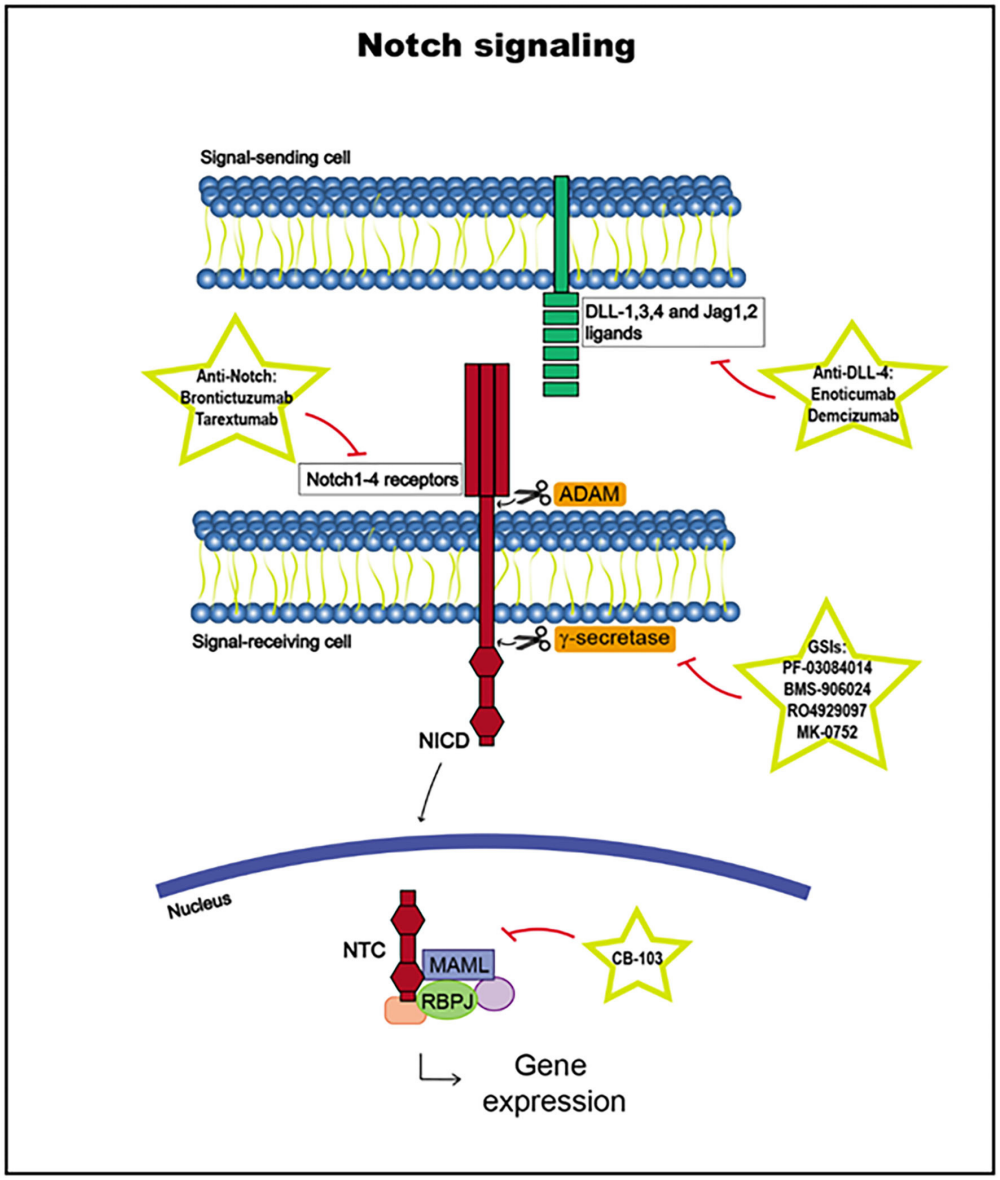
Notch signaling pathway, including targets and drugs that could be employed to inhibit it.

Notch receptor activation begins with the binding of a Notch ligand expressed in a neighboring cell upon the release of the Notch receptor negative regulatory region (NRR). The Notch ligand, which has been previously ubiquitinated in the cytoplasmic tail by E3 ligases Neuralized or Mind Bomb to trigger endocytosis, induces a conformational change in the receptor, exposing cleavage sites for ADAM metalloproteases to access to cleave the extracellular receptor domain. Then, the transmembrane domain is cleaved by γ-secretase, releasing the Notch intracellular domain (NICD). The NICD is translocated to the nucleus and, together with the DNA-binding factor RBPJ and coactivators in the Mastermind-like (MAML) family, it forms the Notch transcription complex (NTC). NTC binds to Notch regulatory elements (NREs), and transcriptional coregulators are recruited, activating the transcription of Notch target genes, such as *HES* and *HEY* (3, 142, 145, 152, 153) (Figure 4).

The Notch signaling pathway is highly regulated, and its target genes vary according to different epigenetic contexts, such as different cell subtypes, changing the cellular outcomes. Indeed, NICD function is influenced by other signaling routes and undergoes many posttranslational modifications at different sites, with a variety of consequences, which have not yet been fully elucidated. Notch receptor expression is also closely regulated in normal cells. Notch1 upregulation is crucial in the early stages of T cell development, whereas its downregulation in later stages is important to prevent the transformation in this lineage. Furthermore, there are other levels of regulation, such as inhibitory feedback loops or RBPJ binding to transcriptional repressors, explaining why deregulation of this pathway can lead to different pathologies, such as cancer development (142, 145, 153, 154).

### Cancer Stem Cells

Loss of balance in the Notch signaling pathway can lead to tumor formation. Indeed, Notch has been implicated in all of the hallmarks of cancers, including cell growth and survival, the EMT, angiogenesis, and/or metastasis. The most frequent alterations have been found in Notch receptor genes. Notch might act as an oncogene or a tumor suppressor, depending on the context and/or the tumor type (140, 142, 145, 149, 155). Interestingly, in some cancers, such as head and neck squamous cell carcinoma (HNSCC), Notch seems to have a bimodal role, as it might function as an oncogene or a tumor suppressor gene (149).

The Notch pathway can be deregulated by mutational activation or inactivation, overexpression, posttranslational modifications or epigenetic alterations (156). In T cell acute lymphoblastic leukemia, translocation 7; 9 produces a fusion gene consisting of the end of Notch1 and enhancer elements or the TCRβ gene, causing a the removal of the NRR receptor activation inhibitor, and ligand-independent activation of the Notch pathway. Other tumors are characterized by mutations in the Notch PEST domain (C-terminal domain), such as B cell tumors (chronic lymphocytic leukemia, splenic marginal zone lymphoma, etc.) or basal-like breast cancer (142, 157). In these cases, among others, such as cancer of the pancreas, prostate, and lung, hepatocellular cancer, esophageal tumors, and HNSCC (149), Notch functions as an oncogene. On the other hand, several tumors present with mutations in the Notch N-terminal domain, producing a loss of function, such as squamous cell carcinomas (skin, head and neck, esophagus and lung) and small cell lung cancers, urothelial carcinomas, and low-grade gliomas; in these cases, Notch acts as a tumor suppressor (142, 149).

Notch has also been associated with stemness maintenance, important in adult organisms and in pathologies such as cancer. In several cell types, Notch activation seems to maintain or promote expansion of stem cell pools, especially in solid tumors such as glioblastoma, hepatocellular carcinoma, ovarian cancer, breast cancer, and HNSCC. Notch inhibitors can decrease stemness marker expression and sensitize tumor cells to chemo- and radiotherapy (142, 149, 153, 155, 157–160).

Notch signaling mediates the interactions between cancer stem cells and tumor microenvironment cells (endothelial, immune, and mesenchymal cells). The Notch pathway is activated in tumor cells by ligands localized in the vascular niche. Specifically, Jag1 is the main ligand that plays this role. For example, in B cell lymphomas, this interaction induces aggressive behavior and resistance to chemotherapy. In other tumors, Jag1 promotes cancer stem cell self-renewal, proliferation and stemness maintenance. Jag1 can eventually activate the transcription of several genes, such as Klf4 in head and neck cancer cell lines, inducing a stem cell phenotype and chemotherapy resistance (145).

### Therapeutic Targets and Drugs

Among the signaling pathways that are activated in cancer cells, the Notch signaling pathway is among the most upregulated, and it is implicated in cancer metastasis, angiogenesis and CSC self-renewal, making it is an important target in cancer therapy (144).

There are two groups of inhibitors that have been developed to target the Notch signaling pathway: γ-secretase inhibitors (GSIs) and monoclonal antibodies against Notch ligand-receptor interactions (mAbs). These therapeutic strategies can be effective in combination with conventional therapies to treat cancer patients with promising results. Furthermore, GSIs and mAbs have been demonstrated to be useful as single agents in the early stage of several tumors, such as thyroid, lung, intracranial and colorectal cancers, and sarcomas. However, Notch signaling is also crucial for normal tissue homeostasis; therefore, its inhibition can induce serious side effects, especially GSIs, such as gastrointestinal toxicity, diarrhea, hepatotoxicity, and nephrotoxicity (3, 140, 144, 149, 161). For this reason, these inhibitors are dose limiting and are administered intermittently (144).

#### γ-Secretase Inhibitors (GSIs)

γ-Secretase is the enzyme critical for releasing the Notch intracellular domain (NICD) and activating the signaling pathway. Therefore, this enzyme may offer promising results as an inhibiter of the Notch pathway. GSIs constituted the first class of inhibitors developed to be used in cancer patients, and more than 100 GSIs have been developed to date (162).

There are many GSIs currently in clinical trials in several cancer types, as single agents or in combination. For example, PF-03084014 is a small reversible and non-competitive GSI that is being studied alone or in combination in several cancer subtypes (163, 164), but currently, its clinical development has focused on desmoid tumors (phase II/III) (165). BMS-906024 is being studied in relapsed T-ALL and T cell lymphoblastic lymphoma (phase I) (166), as well as adenoid cystic adenocarcinoma (phase II), with promising results (3). Another GSI, MK-0752, is a non-competitive oral inhibitor that is being tested for use against recurrent central nervous system tumors, advanced breast cancer and T-ALL (phase I) and in combination with docetaxel or gemcitabine for use in locally advanced or metastatic breast and pancreatic cancer, respectively (phase I/II) (3, 144, 167, 168). RO4929097 has been studied as a single agent in renal cell carcinoma, metastatic pancreatic cancer, and non-small cell lung cancer relapses (phase II) and in combination with temsirolimus, cediranib, gemcitabine, or bevacizumab in different solid tumors (phase I) (3, 144, 169–171).

All these GSIs have been demonstrated to promote the inhibition of the Notch pathway, tumor growth, angiogenesis, stem cell marker expression, and metastasis, etc. (3, 140, 144). However, some of them, such as MK-0752 or RO4929097, are not being developed for use in active clinical trials because of the limited efficacy observed (3) (Table 1).

#### Monoclonal Antibodies (mAbs)

Several monoclonal antibodies have been developed to inhibit Notch signaling as in inhibitors of ligands (DLL-4) and receptors (Notch1–3). They impair ligand-receptor interactions or extracellular domain conformational changes, which are crucial for exposing Notch receptor cleavage sites (140, 144).

DLL-4 is one of the ligands that binds Notch receptors and is involved in growth control, stem cell renewal and development. The deletion of its gene has lethal consequences in the vasculature, and its overexpression has been found in cancer cells and tumor vasculature. Furthermore, DLL-4 inhibition impairs the formation of functional capillaries, leading to aberrant angiogenesis and promoting direct effects that inhibit Notch signaling in tumor cells (172–175).

Enoticumab is a humanized mAb against DLL-4 that seems to have a reasonable safety profile and efficacy in advanced solid cancers, such as ovarian carcinoma (phase I) but is no longer in clinical development. Demcizumab is a DLL-4 antibody that is being studied with gemcitabine in pancreatic cancer, with FOLFIRI in colorectal cancer (phase I) and with carboplatin and pemetrexed in non-squamous NSCLC (phase II), but it did not improve efficacy; therefore, it not in clinical development (3, 140, 144).

Monoclonal antibodies against Notch receptors have also been studied. There are two groups of monoclonal antibodies: those directed to the NRR domain, preventing the conformational change necessary to activate Notch signaling, and those targeted to the EGF-repeat region in Notch receptors to impede the ligand-receptor interaction. Both groups promote substantial downregulation of Notch1 signaling (161, 176).

Tarextumab is a humanized monoclonal antibody against Notch2 and 3 that inhibits Notch signaling as a single agent or in combination with chemotherapeutic agents in epithelial cancers, such as breast, small cell lung, ovarian, and pancreatic cancers (phase I/II). It can inhibit tumor cell proliferation, reduce the number of CSCs and prolong the time before tumors recur after chemotherapy. However, the clinical trials did not show survival improvement; therefore, clinical development has been discontinued (3, 140, 144). Brontictuzumab is an anti-Notch1 mAb that is being studied in hematological malignances and advanced solid tumors (phase I) (177, 178) (Table 1).

#### Other Therapeutic Targets

The DLL-3 Notch ligand is highly expressed in neuroendocrine carcinomas, such as most small cell lung cancers. DLL-3 can promote tumorigenesis through Notch signaling pathway inhibition (in contrast to DLL-4). Therefore, DLL-3 is being studied as a therapeutic target in these cancers. Some other approaches include co-administering AMG 757, a bispecific T cell engager; AMG 119, a chimeric antigen receptor (CAR) T cell (phase I); or as an anti-DLL3 antibody-drug conjugate (ADC), rovalpituzumab tesirine (phase II) (3, 179, 180).

Another strategy involves inhibiting the formation of the Notch transcriptional complex, which acts downstream of abnormal Notch receptor activation. CB-103 is an oral pan-Notch inhibitor that belongs to a novel class of small molecules that target the Notch transcriptional complex, inhibiting the expression of target genes. CB-103 is being studied in phase I/IIa clinical trials for patients with advanced-stage solid tumors or hematological malignancies (181) (Table 1).

## Sonic Hedgehog Pathway

The Sonic Hedgehog pathway plays a role in embryogenesis and brain development. In adults, it is usually inhibited, although it participates in the maintenance of somatic stem cells and pluripotent cells of many organs, tissue repair, and regeneration of several epithelial cells (182–186). Furthermore, there is evidence that the Hedgehog pathway (Hh) is deregulated in various cancer types, such as pancreatic, gastric, prostate, and esophageal cancer (187–189). The activation of the Hh signaling pathway may have a variety of effects involving cell proliferation, cell fate determination, the epithelial-to-mesenchymal transition, and cell motility or adhesion. Therefore, the deregulated activation of this pathway may lead to the development of tumors or resistance to treatment (190).

There are two different mechanisms by which Hh signaling activated: ligand-receptor binding, known as the canonical pathway, or as a consequence of the activation of another downstream member of the signaling pathway, known as the non-canonical pathway (191).

### Canonical

The main receptor in the Hh signaling pathway is Patched (Ptch1), which localized to the base of the primary cilia (PCs), structures that protrude from the cell membrane to sense a variety of stimuli (192). The main ligand that binds Ptch1 is Sonic Hedgehog (SHh), but there are two other ligands: Indian Hedgehog (IHh) and Desert Hedgehog (DHh).

In the absence of Hh binding, Ptch1 does not translocate with the transmembrane protein Smoothened (SMO) to the PCs. When Hh binds to Ptch1, Ptch1 is internalized and degraded, causing the accumulation of SMO in the PC, and as a consequence, the signaling cascade is initiated through a complex formed by kinesin protein (Kif7), suppressor of fused (SUFU) and full-length glioma-associated oncogene (GliFL). This pathway eventually leads to the translocation of the Gli transcription factor to the nucleus.

There are three Gli proteins: Gli1, Gli2, and Gli3. Gli1 is a transcriptional activator, whereas Gli2 and Gli3 could act as activators or repressors. However, Gli2 acts mainly as an activator, and Gli3 acts as a repressor.

**Figure 5:**
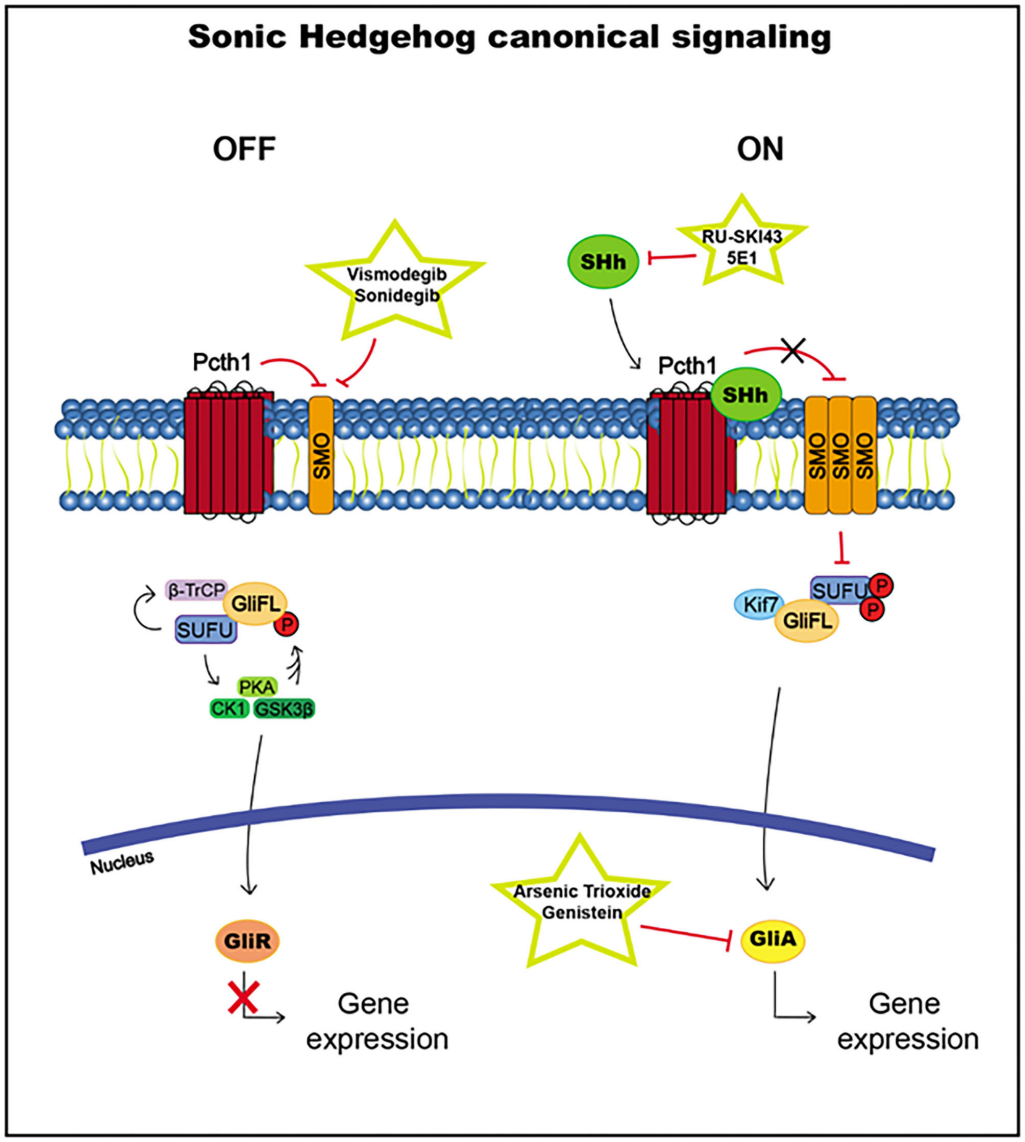
Sonic Hedgehog canonical signaling, including targets and drugs that could be employed to inhibit it. **(Left)** inactive signaling pathway. **(Right)** active signaling pathway.

SUFU usually inhibits Gli transcription factor translocation to the nucleus by direct binding. SUFU activates the phosphorylation by glycogen synthase kinase 3 beta (GSK3β), casein kinase I (CK1), and protein kinase A (PKA) and promotes the recruitment of β-transducin repeat-containing protein (β-TrCP), which causes the transformation of Gli2 and Gli3 into the Gli repressors Gli2R and Gli3R, which are translocated to the nucleus to inhibit the transcription of Hh pathway target genes (186, 190, 193) (Figure 5).

When the signaling pathway is activated by the Hh ligand, the accumulation of SMO leads to the hyperphosphorylation of SUFU, releasing Gli proteins, which translocate to the nucleus and activate the transcription of the target genes Ptch1 and Gli1 (186, 193) (Figure 5).

### Non-canonical

The non-canonical signaling pathway is independent of Gli proteins but is not yet fully understood. In the type I non-canonical pathway, SMO appears to be critical for modulating Ca^2+^ and the actin cytoskeleton, whereas SMO does not participate in the type II non-canonical signaling pathway, which may enable cell proliferation and survival (194).

### Cancer Stem Cells

The cancer stem cell (CSC) model explains tumor heterogeneity through the presence of a small group of cells with unlimited self-renewal capacity and the potential to regenerate all cell types in the entire tumor. They seem to be critical for therapy resistance and relapses. The Hh signaling pathway, as well as other signaling pathways, has been implicated in the maintenance of CSCs (190, 195, 196).

The mechanisms by which the Hh signaling pathway can be activated are mutations in Hh signaling members or the deregulation of Hh ligand release. Furthermore, the Hh signaling pathway may participate in the formation of CSCs.

A variety of members of the Hh pathway have been found to be affected by mutations in human cancers, such as the inactivating mutations of Patch1 or SUFU or the activating alterations of SMO, Gli1, and Gli2, which lead to signaling pathway activation independently of ligand binding. Patch1 loss of heterozygosity is usually found in Gorlin syndrome or nervoid basal cell carcinoma syndrome. Furthermore, alterations to the Hh pathway in combination with tumor suppressor mutations are able to generate other sporadic tumors, such as skin, medulloblastoma, gastric, and rhabdomyosarcoma tumors (190, 197–199).

In other cases, the Hh signaling pathway is stimulated by a deregulation in ligand release, which could be autocrine (from the tumor cell to itself, as it seems to occur in gliomas), paracrine (from the tumor cell to the stroma or from the stroma to the tumor cell, as it may happen in multiple myeloma, which has been studied to a lesser extent) or both, as it might take place in esophageal or gastric cancers (182, 190, 195, 196, 199). It is thought that tumor cells can release ligands that may stimulate the Hh signaling pathway in stromal cells via paracrine signaling and then promote a supportive microenvironment for the tumor (190).

Hh signaling pathways have been demonstrated to participate in the formation and maintenance of cancer stem cells in a variety of tumors, such as hematological malignancies and gastric, pancreatic, prostate, and lung cancers. The Hh signaling pathway is able to activate the transcription of key genes that contribute to the stem cell phenotype, such as Nanog, Oct4, Sox2, and Bmi1. Targeting this pathway in CSCs may be a promising strategy to reduce tumor growth, relapse, and metastasis (200–202).

### Therapeutic Targets and Drugs

Tumors that present with mutations in signaling components may be ameliorated by some Hh pathway inhibitors, but the efficacy of these treatments is dependent on the level of alterations in the signaling pathway. In addition, ligand-dependent tumors may be treated by inhibitors of the Hh signaling cascade that are directed against any of its signaling components, regardless of the level in the route (190).

Among all proteins that may take part in the signaling cascade, SMO and Gli transcription factors are the main targets on which current research is focused. Although there are currently many SMO inhibitors, spontaneous mutations can arise as a consequence of the treatment, which may cause drug resistance (203).

#### SMO Inhibitors

Cyclopamine, a natural alkaloid from *Veratrum californicum*, was the first SMO inhibitor, but the significant side effects in mice did not allow it to be used in humans. Vismodegib, a second-generation cyclopamine derivative approved by the FDA in 2012, is being used in metastatic basal cell carcinoma (BCC) treatment and in recurrent locally advanced BCC, which are not candidates for surgery or radiotherapy. More than 85% of BCC patients have constitutive activation of the SHH pathway, most of which are due to a mutation in PCHT1. Vismodegib binds to SMO and inhibits its function, but continuous exposure can induce mutations in SMO, promoting drug resistance. Furthermore, vismodegib is being studied in many clinical trials in a variety of human cancers, such as breast cancer (204–206).

Sonidegib is another SMO antagonist, approved in 2015 by the FDA, that is used in the treatment of patients with locally advanced BCC that recurred disease after surgery or radiotherapy or patients who are not able to receive surgery or radiation treatments. Sonidegib, similar to vismodegib, binds SMO in the “drug binding pocket,” and mutations at this site lead to resistance (205, 206). Sonidegib and Vismodegib are teratogen, as other Sonic Hedgehog pathway targeting drugs, due to the role of this signaling route in embryogenesis (206).

There are other drugs that are currently in clinical trials for different cancer types, such as Saridegib (phase II), BMS-833923 (phase II), glasdegib (phase II), taladegib (phase II), or TAK-441 (phase I), and they have shown promising results in preclinical models (205, 206). TAK-441 has shown efficacy in cells that have developed adaptive mutations in SMO; therefore, it may be relevant for patients with drug resistance (206) (Table 1).

#### GLI Inhibitors

Arsenic trioxide, a Gli inhibitor approved by the FDA for the treatment of acute promyelocytic leukemia, binds directly to Gli1 and Gli2, among other actions, causing the inhibition of their activity (205, 206). It has been shown to affect the viability of cancer stem cells in pancreatic and prostate cancer (in the cancer-initiating cells). This drug is being studied in phase I–IV clinical trials for solid tumors and hematological malignancies. Arsenic trioxide could imply some cardiac side effects such as QT interval elongation or tachycardia, as well as circulatory, gastrointestinal and respiratory disorders (205–207).

Genistein (phase I and II), an isoflavone isolated from *Genista tinctoria*, has been shown to inhibit Gli1, causing the growth suppression of different tumors and the cancer stem niche (206, 208) (Table 1).

#### SHh Inhibitors

Inhibitors of SHh, which is the most important ligand, have not yet been approved for use in the clinic, but they have been shown to effectively inhibit the SHh pathway in mice. Some of these are SHh monoclonal antibody 5E1, or RU-SKI43, which inhibits the SHHat enzyme, critical for catalyzing the binding of palmitate to SHh, one of the final steps in SHh synthesis (205, 206) (Table 1).

#### Other Therapeutic Targets

A truncated isoform of the Gli1 transcription factor, which indicates a gain of function, has been discovered: tGli1. This isoform has been detected exclusively in tumor cells, not in normal cells, and promotes tumorigenesis to a greater extent than does Gli1, which makes it a promising drug target. There is no drug directed specifically against tGli1, but it may be possible to target the genes activated by tGli1, such as CD24, VEGF-A, VEGFR2, or HPA1, and inhibit their expression. A phase I clinical trial is testing a CD24 monoclonal antibody. Furthermore, some heparanase (HPA1) inhibitors, such as PI-88 or PG545, are being studied as antiangiogenic anticancer drugs (PI-88 in phase III and PG545 in phase I). Finally, there are several antiangiogenic drugs approved by the FDA and directed against VEGF-A and VEGFR, such as bevacizumab, Ziv-aflibercept, and sorafenib (205).

## Hippo Pathway

**Figure 6:**
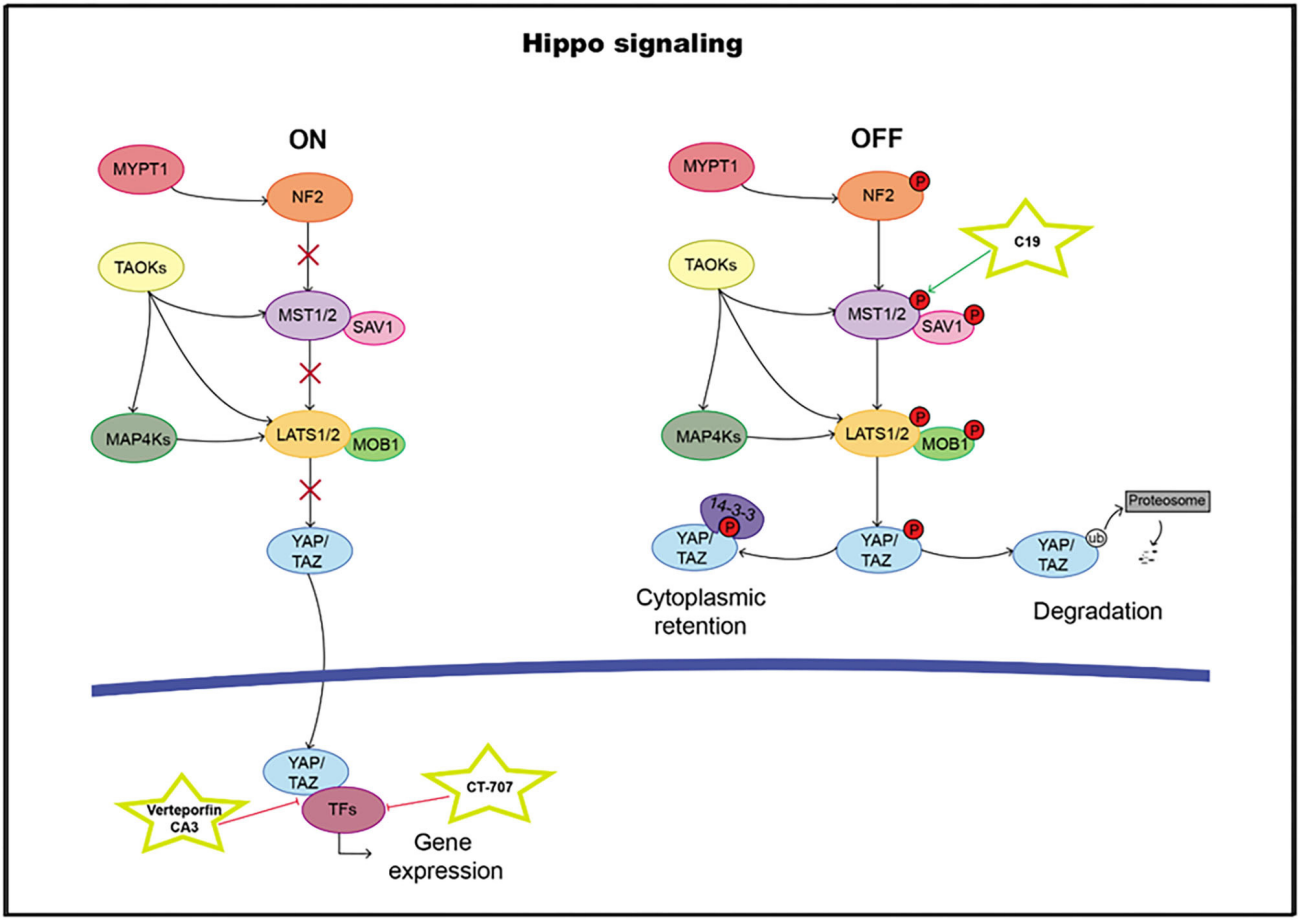
Hippo pathway including targets and drugs that could be employed to inhibit it. **(Left)** active signaling pathway. **(Right)** inactive signaling pathway.

The highly evolutionarily conserved Hippo signaling pathway regulates biological processes, such as survival, differentiation, cellular proliferation, fate determination, organ size, or tissue homeostasis (209). The core pathway consists of a kinase cassette that is composed of MST1/2 and LATS1/2 (210). In addition to MST1/2, MAP4K, and TAOK also directly phosphorylate LATS1/2 (211–214). NF2 is critical for pathway activation through the phosphorylation of MST1/2. The major target of the Hippo pathway is YAP and its paralog transcriptional coactivator TAZ. Phosphorylation of YAP and TAZ leads to their sequestration in the cytoplasm by 14-3-3 proteins and ubiquitination-dependent proteosomal degradation (215). In the nucleus, YAP/TAZ it can bind and regulate a family of sequence-specific transcription factors called TEA DNA-binding proteins (TEAD1–4) that modulate genes such as CTGF, CRY61, BIRC5, ANKRD1, and AXL, involved in proliferation and survival. In addition to TEADs, the YAP/TAZ complex also cooperates with RUNX1 and 2, TBX5, and SMADs, among others (216–219) (Figure 6). High TEAD expression levels have been correlated with poor clinical outcomes, and therefore, it can serve as a prognostic marker in many solid tumor types (220–231).

### Cancer Stem Cells

Hippo pathway effectors activated by YAP/TAZ have been shown to induce cancer stem cell (CSC) properties in a wide range of human cancers, including osteosarcoma, glioblastoma, and chemoresistant breast cancer (232, 233). Moreover, breast cancer tissues with a high content of CSCs show a gene expression profile that overlaps with YAP/TAZ-induced gene expression, and breast CSCs with a CD44+/CD24– phenotype have a relatively high expression of TAZ (234).

YAP/TAZ are known to promote other properties of CSCs, such as the epithelial-to-mesenchymal transition (EMT) and metastasis, via activation of TEAD transcription factors in different tumors, including breast cancer and melanoma (235–241). In the context of these tumors, TEAD activation leads to the disruption of cell–cell junctions, mesenchymal gene expression, increased cell migration, anoikis resistance and cell invasion. TEAD specifically regulates genes such as ZEB1, ZEB2, DNp63, and Slug, which induce an increase in the progression and metastatic potential of tumors such as squamous cell carcinoma, breast cancer, and small-cell lung carcinoma (NSCLC) (242–244). Interestingly, YAP expression was critical for the progression of various KRAS-driven cancers, and YAP/KRAS converged on FOS to promote the EMT, which contributed to oncogenic KRAS oncogenic addiction (245).

It has also been proven that the activation of YAP/TAZ confers resistance to chemotherapy in cancer cells, in part because of the CSC characteristics acquired by the cells. YAP/TAZ has been linked to castration resistance in prostate cancer and paclitaxel and doxorubicin resistance in breast cancer (234, 246, 247). Moreover, different studies suggest that Hippo pathway activation promotes cancer cell survival in the presence of DNA-damaging agents such as UV exposure, radiation, cisplatin, Taxol, and fluorouracil (5-FU) in a wide number of tumor types (248–251).

YAP/TAZ, in conjunction with their target genes of secreted ligands, also promote resistance to targeted therapies, such as the drugs targeting RAF and MEK and BRAF inhibitors and receptor tyrosine inhibitors, such as gefitinib (252–258).

The relevance of the Hippo pathway in cancer and, more specifically, in the biology of CSCs has already been demonstrated in multiple publications. Therefore, the Hippo pathway is currently being studied as an interesting target for use in developing targeted therapies for different types of tumors. There are some approved compounds able to regulate this pathway; however, they have shown to have some bioavailability or toxicity problems. Consequently, extensive effort is being made in the research of new drugs to address these issues.

### Therapeutic Targets and Drugs

Regarding therapeutic opportunities for the Hippo pathway, YAP inhibition has been shown to produce promising therapeutic effects in different types of tumors, such as NSCLC, breast cancer and colorectal cancer (259–262). Additionally, the regulation of upstream or downstream molecules of this pathway also show antitumoral effects. However, the inhibition of the upstream regulators of YAP might serve to increase transcriptional activity and thus be counterproductive. This result makes the inhibition of the YAP/TAZ interaction and the suppression of their binding to their targets the most appealing strategies.

Verteporfin, an FDA-approved drug, was identified to inhibit the interaction between YAP and TEAD (263, 264). Therefore, it can decrease the expression of Hippo target genes; however, it has shown low solubility and stability, unfavorable pharmacokinetics, and rapid clearance; complicating its clinical use. Moreover, this compound lacks tumor specificity and have serious Hippo-independent effects, and thus, may originate adverse effects in healthy tissues (265–267). In addition, its mode of inhibition and mechanism of interaction with YAP remain unknown. Considering all, verteporfin might not be the most promising drug, nevertheless, some studies have shown that the loading of verteporfin into microparticles improves its specificity and pharmacokinetics, making it more suitable as a treatment (267–269). Similarly, the CA3 compound has been reported as a modulator of YAP/TEAD transcriptional activity through the inhibition of YAP, but it has drawbacks similar to those of verteporfin (270, 271).

Another possibility for YAP/TEAD inhibition is the lipid pocket at the core of the TEAD family, which is essential for TEAD folding, stability, and YAP binding (272–274). Some compounds targeting this domain in cell assays, such as flufenamic acid or chloromethyl ketone moieties, have been shown to inhibit cell proliferation and several Hippo pathway responsive genes. However, the underlying mechanism remains unclear because, in some cases, YAP/TAZ binding remains unchanged (275, 276). In recent studies, a cyclic YAP-like peptide has been shown to block the YAP-TEAD interaction through competition with endogenous YAP (277). However, this peptide has not yet been converted into a cellularly active compound. “Super-TDU,” a peptide mimicking VGLL4, has also been reported to compete with YAP binding to TEADs. Its mechanism is based on the binding of vestigial-like proteins (VGLL) to TEADs, and it has led to the reduced growth of gastric cells *in vitro* and *in vivo* (278). All these antagonistic compounds offer promising strategies; however, their clinical viability and applicability remain unproven.

Due to YAP druggability problems, it has been proposed to target its posttranscriptional modifications, which are critical for its oncogenic properties. In this context, some already approved drugs, including metformin, statins, dasatinib, pazopanib, and nicotinamide (NAM), and some agents being tested in clinical trials, such as the multikinase inhibitor CT-707, have been reported to decrease YAP activation through the regulation of posttranscriptional modifications (279, 280). These findings suggest that they might be plausible cancer therapies, although many more studies are needed.

Another potential approach to inhibiting the Hippo pathway is through the activation of MST and LATS kinases, which phosphorylate and inactivate YAP (281). The small-molecule compound C19 has shown the ability to activate MST and therefore to suppress melanoma cell growth in a mouse xenograft model (282). Moreover, metformin and statins have been known to activate LATS and inhibit YAP activity (283). However, their applicability as cancer therapeutic agents is still controversial and requires more clinical trials (284, 285).

Finally, the downregulation of Hippo pathway regulators is also possible at the transcriptional level. The combination of some epigenetic modulators, including I-BET151 and panobinostat, has been shown to induce the synergistic downregulation of the AKT and Hippo pathways in melanoma cell lines without binding to the cytoplasmic proteins of these pathways (286). Nevertheless, compounds regulating the epigenome show some drawbacks, for instance, off-target effects due to their lack of specificity, low stability and sustainability, and significant toxicity in normal cells (287).

Altogether, there are significant advances in the field of developing targeted therapies for the Hippo pathway. They could be a priceless weapon in the fight against cancer; nevertheless, we need more research in this area. It is important to better understand the mechanism of action of these compounds and to develop clinical trials to test their secondary effects and their applicability in cancer patients (Table 1).

## TLR Pathway

Toll-like receptors (TLRs) belong to the pathogen recognition receptor (PRR) group in the innate immune system. They recognize exogenous ligands from invading pathogens (pathogen-associated molecular patterns or PAMPs) and endogenous ligands released from damaged host cells (damage-associated molecular patterns or DAMPs) (288–290). Ten TLRs are found in both human immune cells (T-cell and B-cell subsets, macrophages, and dendritic cells) and non-immune cells (epithelial cells and fibroblasts). Some of these TLRs are localized in intracellular vesicles, for instance, TLR3, TLR7, TLR8, and TLR9, while others are localized on the cell surface (291). TLRs are type I glycoproteins that share some common structural domains: an extracellular domain containing multiple leucine-rich repeats that enable the recognition of the ligand, a transmembrane region, and a highly conserved intracellular Toll-interleukin 1 (IL-1) receptor domain (TIR) necessary for signal transduction (292–294).

**Figure 7:**
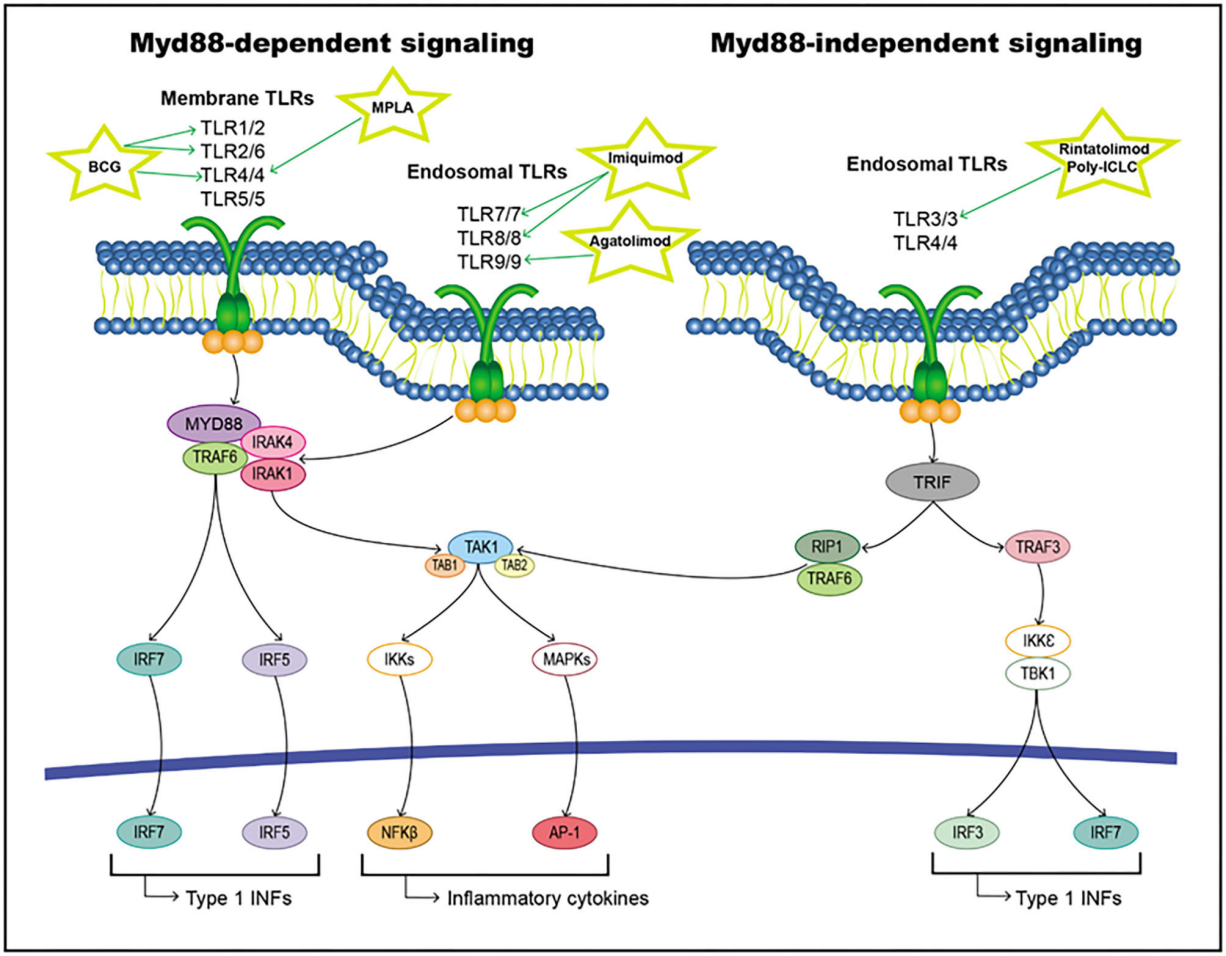
TLRs pathway including targets and drugs that could be employed to inhibit it. **(Left)** Myd88-dependent signaling. **(Right)** Myd88-independent signaling.

When a ligand binds to the extracellular domain of a TLR, it induces a conformational change in the receptor allowing for its homodimerization, and therefore, the binding of their TIR domains and the recruitment of different intracellular adaptor molecules. These adaptor proteins include Myd88, TRIF/TICAM-1, TIRAP/Mal, TIRP/TRAM, and SRAM (295, 296). All TLRs except TLR3 initiate signaling through MyD88 (the classical inflammatory pathway), forming a Myd88/IRAK1/IRAK4/TRAF6 axis that activates TAK1. This pathway triggers the activation of transcription factors, such as NF-κB, AP-1, and IFN regulatory factors (IRFs) (297–300). On the other hand, TLR3 and TLR4 can induce signaling through TRIF/TIRAM adaptor molecules instead of Myd88, leading to the activation of IRFs (301). All these activated transcription factors are translocated to the nucleus and interact with their target genes, including inflammatory cytokines, chemokines, and type I interferon (IFNs) (Figure 7).

Alterations in TLR signaling can have both antitumoral and protumoral effects on carcinogenesis and tumor progression. These effects depend on the TLR class, the cell type and the signaling pathway that is triggered in those cells. For instance, TLR agonists have been used as adjuvants in anticancer vaccines to stimulate immune cells to fight the tumor (302, 303). However, TLR expression in some cancer and immune cells is related to the activation of genes related to tumor progression and thus to tumor growth (304). Due to this double action of TLR signaling, it is important to study the role of each type of TLR in cancer individually and to always consider the origin of the tumor being investigated. Additionally, various cells in the tumor and its microenvironment can have different TLR expression patterns, and therefore, they will react differently to TLR modulation (305, 306).

### Cancer Stem Cells

Despite their involvement in immunity against tumor invasion, the TLR signaling pathway with cancer stem cell properties have been found in different tumor types. For example, TLR2 activation in epithelial ovarian cancer has been shown to enhance a proinflammatory environment and thus to increase cell self-renewal through the upregulation of stem cell-associated genes (307). Similarly, targeting TLR3 with an agonist in breast cancer cells led to the expression of stem-associated genes, including OCT3/4, NANOG, and SOX2, because of the activation of β-catenin and NF-κB signaling pathways (308). Additionally, in murine models of hepatocellular carcinoma, TLR4 expression was associated with stem-like properties, including the invasion and migration of cells. TLR4 stimulation is suggested to enhance the emergence of stem-like cancer cells through Nanog and STAT3, which cooperate to activate the metastasis regulator TWIST1 (309). Accordingly, there was a strong correlation between TLR4 expression and poor prognosis for patients (310, 311). Moreover, in breast cancer, TLR4 activation has resulted in enhanced stemness and tumorigenicity of cells. It has also been linked to poor prognosis and an increased relapse rate in patients (312). Nevertheless, the downregulation of TLR4 in glioblastoma CSCs has been observed. This result may be considered a mechanism by which these CSCs escape immune surveillance and is thought to be related to an increase in retinoblastoma-binding protein 5 (305).

In the case of TLR7, CD133+ cells in colorectal cancer patients showed increased expression of this marker, which was associated with a poor prognosis (313). Similarly, it has been suggested that TLR9 is able to promote the stem-like prostate cancer cell phenotype through NF-κB and STAT3 upregulation of stem-related genes, including NKX3.1, KLF-4, BMI-1, and COL1A1 (314). It has also been demonstrated that the upregulation of TLR9 in glioma cancer stem-like cells is able to activate STAT3 and thus maintain the quiescent state of tumor-repopulating cells (315).

All this information suggests a feedback loop of inflammation and stemness in tumor cells. The presence of extrinsic stimuli in the tumor microenvironment can activate proinflammatory pathways, for instance, TLR signaling. This activation leads to the upregulation of genes related to stemness and the epithelial-to-mesenchymal transition (EMT), which are able to induce and maintain the dedifferentiation of cells, converting them to CSCs (115, 316–319). On the other hand, CSCs constitutively exhibit deregulation in the expression of NF-kB, increasing the levels of inflammation within the tumor (320). This positive feedback loop might promote malignancy and resistance to treatments. Therefore, targeting TLR signaling may be a promising strategy for reducing CSC expansion and, thus, tumor progression.

### Therapeutic Targets and Drugs

Targeting TLRs represents a promising strategy for tumor immunotherapy due to their ability to activate innate immunity and even stimulate adaptative responses for long-lasting defense against tumor antigens. Furthermore, in some cases, the activation of certain TLRs, such as TLR2 or TLR4, can have direct antitumoral effects on cancer cells. These TLRs have been used in different approaches; for example, they have been used as adjuvants for cancer vaccines or in combination with radiotherapy, chemotherapy and other immunotherapies. However, when targeting this pathway, it is important to consider that the same TLR can have different behavior, either protumoral or antitumoral, depending on the tumor type.

#### Treatments Based on TLR Agonism

There are multiple cancer treatments based on activating the immune response through different TLR receptors. A large number of compounds have been developed and used as monotherapy or in combination with other strategies to treat diverse types of tumors. Some molecules targeting this pathway have already been approved by the Food and Drug Administration (FDA) and are currently being used to treat patients. Many more are being tested in preclinical and clinical trials and might be in the clinic in the near future.

Bacillus Calmette-Guerin (BCG) is an FDA-approved treatment that activates TLR2/4 through its cell wall and TLR9 through its bacterial DNA (321, 322). It is the standard of care for patients with carcinoma *in situ* or urinary bladder cancer that has not invaded muscle (323). However, it still shows a 50% risk of failure (324) and the difficulty of its manufacturing process and its increasing demand have created a shortage of this treatment (325). Agents similar to BCG and their combination with other therapies are being tested in multiple clinical trials for different tumor types, such as colorectal cancer (326, 327), melanoma (328–330), and small cell lung cancer (331). Other bacteria that activate TLR2, such as *Mycobacterium indicus pranii* (Immuvac, CADI-05), are also being studied. CADI-05 is an approved treatment for leprosy, and interestingly, it has been shown to reduce myeloma and thymoma tumor size in murine models (332, 333). In addition, monophoryl lipid A (MPLA), currently used as a synthetic adjuvant in vaccines, is a derivative of lipid A and can stimulate TLR4. Some of its analogs are being tested as adjuvants for cancer vaccines, for instance, glucopyranosyl lipid A for skin cancer (334) and AS15 (a combination of MPLA with other immune stimulators) in distinct types of tumors (335, 336). These type of combinations might show a slight increase of mild side effects probability, specially, local injection site reactions (336, 337).

In the case of TLR3, Poly (I:C) is a TLR3 ligand that functions as a potent adjuvant for cancer vaccines (338); however, due to its fast degradation, new alternative agonists are being investigated. Some Poly(I:C) derivatives are promising, for instance, poly-ICLC (Hiltonol®) for solid tumors (339) and rintatolimod (Ampligen®) for fallopian tube, ovarian and brain tumors (NCT03734692 and NCT01312389).

Targeting TLR5 has shown promising effects in murine models, in which flagellin and different nanoparticles have shown antitumoral effects (340–342). A *Salmonella* flagellin derivative, entolimod (CBLB502), is being tested in clinical trials against advanced local and metastatic malignancies (343). Additionally, it has demonstrated radioprotective effects in animal models (344).

The TLR7/8 agonist imiquimod has been approved by the FDA and is currently being used for treating superficial basal cell carcinoma (345, 346). It has also shown efficacy in the treatment of different cutaneous tumors (347–349), and it is being tested in several clinical trials for skin and other malignancies, such as glioma and breast and prostate cancer (350, 351) (NCT01792505, NCT00899574, and NCT02234921). It might cause some local inflammatory reactions and systemic symptoms, including muscle aches, fatigue, and nausea, but, in general terms, it is well-tolerated (352). Resiquimod is another TLR7/8 agonist and has shown a more intense immune response than imiquimod (353). It is being explored in precancerous and malignant skin tumors (354, 355) and in multiple clinical trials as a vaccine adjuvant. Additionally, 852A, a TLR7 agonist, and VTX-2337, a TLR8 agonist, are being examined in clinical trials against different tumor types, such as ovarian, breast, cervical, endometrial, and head and neck cancers (NCT00319748 and NCT01334177).

Finally, CpG oligodeoxynucleotides are agonists of TLR9 and are being tested in several tumor types and in some clinical trials (356–359). For example, Agatolimod (CpG 7909) and is being studied as a vaccine adjuvant and as a monotherapy for various solid and hematological malignancies (360–362). Although the tolerability and safety of TLR9 ligands in monotherapy have been proven in numerous clinical trials, they have shown scarce antitumoral efficacy (363). The combination with other immune modulating compounds can greatly improve CpG ODNs-based strategies. Hence, Agatolimod is also being tested in combination with monophosphoryl lipid A and MAGE-A3 (a melanoma antigen) in phase II clinical analysis (NCT00085189). Furthermore, an alternative TLR9 agonist, SD-101, has been shown to overcome resistance to checkpoint inhibitors and is being investigated in association with these inhibitors in ongoing clinical trials (NCT02521870). With these types of drugs the primary adverse effects are also related to immunostimulation or systemic-flu like symptoms (364). Nevertheless, they could lead to autoimmune disorders if used as long-term treatments (365) (Table 1).

#### Treatments Based on TLR Antagonism

In some cellular locations, TLR antagonism, not TLR activation, is needed. For this purpose, inhibiting treatments are being tested, and some have entered phase I and II clinical trials. Molecules derived from LPS, such as E5564 and CRX-526, and antibodies targeting TLRs, such as OPN305, are able to inhibit TLR signaling and reduce inflammation, but they are not currently being used against cancer (366–369) (NCT02363491). Recently, various studies have shown that the blockade of TLR4 might have antitumoral effects in ovarian, breast and prostate cancer (370–372). In another possible approach, bacteria, and/or gut microbiota are modulated by using probiotics or antibiotics. This approach has been shown to reduce TLR tumor-promoting signals and inflammation in several studies (373–376). However, the use of this technique in cancer is still being developed (Table 1).

#### Future Treatment Opportunities Targeting the TLR Pathway

Immunomodulation in cancer is currently being intensely researched, with some approved innovations showing substantially positive results, such as antibodies targeting T cell-mediated immune checkpoints, adoptive cellular therapies, or cancer vaccines. In this context, numerous trials are combining these novel treatments with TLR agonists to improve their activity (377–380).

Another possible approach using TLR as a clinical cancer treatment is based on targeting its downstream modulators. The MYD88 protein is involved in surface and endosomal TLR pathways and thus is considered an attractive candidate. In addition, some studies suggest that it may be involved in oncogenesis (381) and related to the clinical response to ibrutinib (382, 383). Similarly, IRAK4 is a downstream mediator of the pathway that links multiple TLRs to NF-κB activation. Constitutive activation or increased expression of this gene has been reported for different tumor types and is correlated with chemoresistance (384, 385). Other proteins, including TRAF6 and NF-κB, have been shown to stimulate bortezomib activity in preclinical models (386).

Finally, TLR might serve as a protective agent in cancer treatment. TLR5 agonists have shown chemoprotective and radioprotective effects in numerous studies (344, 387–389). Furthermore, they have been demonstrated to improve the outcome of bone marrow allotransplant and decrease the incidence of graft-vs.-host disease (GVHD) (390, 391).

In summary, TLR-targeted therapies for cancer are currently being successfully used, however, many opportunities remain to develop new compounds to provide better treatment for patients and to minimize the secondary effects of current strategies (Table 1).

## Oxidative Stress and CSCs

The generation of reactive oxygen species (ROS) in a high concentration is detrimental for the cell, due to the fact that oxidative stress promotes DNA, RNA, proteins, and lipids modifications. It is well-known that cancer cells, in consequence of its active metabolism and altered oncogenic and tumor suppressor signaling pathways, are usually characterized by a high ROS level. However, cancer cells are able to adapt and proliferate, becoming resistant to oxidative stress (392–394).

Oxidative stress has been associated with a variety of cancer-related effects: cellular proliferation, apoptosis evasion, invasion, metastasis, or angiogenesis. Different signaling pathways mentioned in this review are implicated in these ROS production outcomes. For instance, ROS could regulate EMT through activation of NFkB signaling pathway, or metastasis through Wnt signaling cascade stimulation, among other signaling routes (392, 395–398).

Aerobic glycolysis is energetically more efficient than anaerobic one, but leads to the generation of a big amount of ROS. Tumor cells accomplish adapting to oxidative stress switching aerobic glycolysis for anaerobic one, independently of oxygen available in the microenvironment. This phenomenon is called Warburg effect, and leads to a low ROS level formation, accompanied by a redox potential increase, through NADPH production (399–402). Furthermore, cancer cells possess higher antioxidant efficiency than non-tumoral cells. In order to counteract the less ATP generation in anaerobic glycolysis, tumor cells considerably increase glucose intake. It has been shown that cancer cells also activate the pentose phosphate pathway (PPP) with a prolonged ROS exposure, in order to acquire NADPH and nucleotides for DNA synthesis and repair (402–406).

Cancer stem cells seem to possess a lower ROS level than non-tumoral stem cells, due to different mechanisms useful to reduce oxidative stress and maintain stem cell properties (404, 407–409). However, there are controversial aspects in the metabolism regulation of CSCs. While several studies show that CSCs often present a high glycolytic metabolism, which preserves them from oxidative stress damaging, others highlight that they could be more dependent on oxidative phosphorylation. Furthermore, CSC could switch from anaerobic glycolysis to PPP in order to obtain a stronger antioxidant potential to maintain stemness. Also, increased PPP has been associated with drug resistance (403, 405, 406, 408, 410, 411).

Due to the dual consequences of an augmented ROS levels, the oxidative stress targeted therapies are under discussion. On the one hand, several well-known anticancer treatments increase ROS levels in cancer cells, in order to reach a threshold which causes the cell death, such as radiation, arsenic trioxide, 5-fluorouracil, or paclitaxel. Another strategies try to decrease ROS scavengers in cancer cells. On the other hand, as it has been mentioned above, ROS is related to several cancer-promoting effects, thus it might be advantageous to reduce ROS levels or increase antioxidant molecules, in order to restore de redox balance in the cell. For these reasons, the efficacy of these anticancer strategies depends on the ROS level in the tumor type and the capacity of the tumor to modulate its metabolism routes (404, 409, 412–419).

## Conclusions

Currently, many observations have indicated the association between the CSC content and clinicopathological characteristics of tumors upon diagnosis. Additionally, CSC populations are resistant to conventional therapies aimed at the bulk of proliferative cells, and they can be enriched in a posttreatment setting. Therefore, targeting stemness pathways may provide promising strategies to actively eradicate tumors and metastasis. There are many pathways that regulate the CSC phenotype and its pluripotency state, providing mechanistic support for acquired drug resistance, including altered metabolism and oxidation states; phenotypic plasticity; increased membrane extrusion pumps and receptors; altered apoptosis, autophagy, and/or cell cycle regulation; different DNA damage, and/or repair responses; epigenetic regulation and/or differential secretion of proteins or non-coding RNA. Some of these stemness pathways have been reviewed here, and their targeting approaches have been described. However, data on their proper response to the inhibitors and accounts of the extent to which they have antitumor efficacy are scarce. In most cases, the inhibitor activity depends on the extent of the molecular dependence on the specific targeted pathway in the CSCs or its ability to avoid targeting mature cells or normal stem cells to prevent undesirable toxicity.

Importantly, the stemness pathways are interconnected to regulate the CSC phenotype and the transition among different pluripotency states. This cross talk among pathways may drive the resistance to single pathway inhibitors and maintain the CSC phenotype. For example, cross talk between PI3K and Notch may contribute to the resistance to therapeutic PI3K inhibitors of breast cancer (420). Additionally, PIM inhibition might not have the same effect as AKT on MEK targets, which may not be effected by PIM single inhibitors *in vivo*. This phenomenon may explain in part the negative results obtained in clinical trials with single-agent therapy.

Other important reason for tumor resistance is based on advanced tumors, in general, containing expanded polyclonal CSC populations, which render them resistant to therapies against single CSC signaling pathways. Therefore, ongoing clinical trials using molecular biomarkers may be used to overcome these challenges. Moreover, the incorporation of CSC-based functional assays (for example, 3D organoids, pluripotency assays) may provide a better view of CSCs and therefore of tumor, eradication using these pathway-based therapeutic strategies.

Despite all of the challenges, some inhibitors for these pathways are currently used as a standard of care for patients, and many others are being tested in phases III and IV clinical trials. A huge effort is being made in order to develop more specific and less toxic compounds to target every single pathway known to be involved in CSC establishment and maintenance. Different drugs, antibodies, vaccines and, even, immunotherapeutic approaches are being assayed for this purpose. Therefore, although more research is needed to undercover additional regulatory elements, we expect to see new molecules targeting these pathways approved by the FDA in the following years.

## Author Contributions

All authors contributed equally to information and writing the work. AC edited the work.

## Conflict of Interest

The authors declare that the research was conducted in the absence of any commercial or financial relationships that could be construed as a potential conflict of interest.
